# Cerebellar contributions to a brainwide network for flexible behavior in mice

**DOI:** 10.1038/s42003-023-04920-0

**Published:** 2023-06-05

**Authors:** Jessica L. Verpeut, Silke Bergeler, Mikhail Kislin, F. William Townes, Ugne Klibaite, Zahra M. Dhanerawala, Austin Hoag, Sanjeev Janarthanan, Caroline Jung, Junuk Lee, Thomas J. Pisano, Kelly M. Seagraves, Joshua W. Shaevitz, Samuel S.-H. Wang

**Affiliations:** 1 Neuroscience Institute, Princeton University, Washington Road, Princeton, NJ 08544 USA; 2 Department of Physics, Princeton University, Princeton, NJ 08544 USA; 3 Lewis-Sigler Institute for Integrative Genomics, Princeton University, Princeton, NJ 08544 USA; 4 Department of Statistics and Data Science, Carnegie Mellon University, Pittsburgh, PA 15213 USA; 5 Department of Organismic and Evolutionary Biology, Harvard University, Cambridge, MA 01451 USA

**Keywords:** Neural circuits, Decision

## Abstract

The cerebellum regulates nonmotor behavior, but the routes of influence are not well characterized. Here we report a necessary role for the posterior cerebellum in guiding a reversal learning task through a network of diencephalic and neocortical structures, and in flexibility of free behavior. After chemogenetic inhibition of lobule VI vermis or hemispheric crus I Purkinje cells, mice could learn a water Y-maze but were impaired in ability to reverse their initial choice. To map targets of perturbation, we imaged c-Fos activation in cleared whole brains using light-sheet microscopy. Reversal learning activated diencephalic and associative neocortical regions. Distinctive subsets of structures were altered by perturbation of lobule VI (including thalamus and habenula) and crus I (including hypothalamus and prelimbic/orbital cortex), and both perturbations influenced anterior cingulate and infralimbic cortex. To identify functional networks, we used correlated variation in c-Fos activation within each group. Lobule VI inactivation weakened within-thalamus correlations, while crus I inactivation divided neocortical activity into sensorimotor and associative subnetworks. In both groups, high-throughput automated analysis of whole-body movement revealed deficiencies in across-day behavioral habituation to an open-field environment. Taken together, these experiments reveal brainwide systems for cerebellar influence that affect multiple flexible responses.

## Abstract (web summary)

Integration of chemogenetics with quantitative behavioral assays and whole brain activity mapping reveal how distinct cerebellar regions influence activity during flexible behavioral learning. Integration of chemogenetics with quantitative behavioral assays and whole brain activity mapping reveal how distinct cerebellar regions influence activity during flexible behavioral learning

## Introduction

The cerebellum is increasingly appreciated for its contributions to flexible behavior. Prominent anatomical pathways between cerebellum and neocortex suggest a role in higher-order processing^1–5^. In humans, insult to the posterior cerebellum results in a clinical cognitive-affective syndrome that includes impairments in executive function, working memory, abstract reasoning, and emotional processing^6,7^. More severe outcomes arise from pediatric cerebellar insult, including a diagnosis of autism, a disorder characterized by inflexibility to the point of emotional distress when routines are violated^8–13^. Taken together, these studies suggest that, like the neocortex, the cerebellum plays a necessary role in flexible behavior and cognitive processing.

Animal experiments have identified specific regions of the cerebellar cortex that support flexible behavior. In vermis lobule VI, a midline posterior structure that is perturbed in autism spectrum disorder^14,15^, inhibition of molecular layer interneurons alters reversal learning, perseverative or repetitive behavior, novelty-seeking, and social preference^16^. Perturbation of rodent crus I, the human homolog^17^ of which is structurally altered in ASD, leads to deficits in social, repetitive, and flexible behaviors^16,18^, and neither perturbation affects gait. Furthermore, inactivation of Purkinje cells in rodent crus I reduces the ability to perform sensory evidence accumulation, a task in which Purkinje cells have been found to encode choices and accumulated evidence^19,20^.

Lobule VI and crus I engage with the forebrain through bidirectional polysynaptic pathways^21^. Purkinje cells in the cerebellar cortex receive input from distal forebrain structures, and transsynaptic tracing in mice has traced the Purkinje cells’ inhibitory output to cerebellar, vestibular, and parabrachial nuclei, which in turn provide excitatory output to the rest of the brain to form a cerebral–thalamic–cerebellar circuit^1–4,22,23,24^. Along these pathways, cerebellar cortex is organized into parasagittal microzones which project in distinctive patterns, so that lobule VI and crus I make different patterns of disynaptic connectivity with thalamic structures^25–28^, and trisynaptic paths to anterior cingulate, infralimbic, premotor, and somatosensory cortex^1–5,28,29^. Each of these cerebellar regions also receives descending input from the neocortex via the pons^30–32^ and inferior olive^33^. These cerebellar regions, therefore, have distinctive routes by which they may influence forebrain processing across many distributed targets.

To interrogate the contribution of the posterior cerebellum to a simple reversal learning task, we monitored mouse behavior and mapped brain-wide patterns of activation after perturbing lobule VI and crus I. First, we chemogenetically perturbed neural activity reversibly in Purkinje cells, the principal output neurons of the cerebellar cortex. Second, we combined a Y-maze learning paradigm with c-Fos mapping to identify brain-wide substrates of reversal learning. Third, we studied expression of the activity-dependent gene product c-Fos using tissue clearing techniques combined with light-sheet microscopy to map across the whole brain without need for tissue sectioning. We analyzed this data to identify individual activated regions and patterns of coactivation. Lastly, we characterized the effects of perturbation on freely-moving mouse behavior in granular detail using machine learning methods for automated tracking of body poses, movements, and actions. Together, these approaches provide a framework for characterizing how the cerebellum influences brainwide functional networks to modulate flexible behavior.

## Results

### Experimental design to reversibly perturb Purkinje cells

**Fig. 1:**
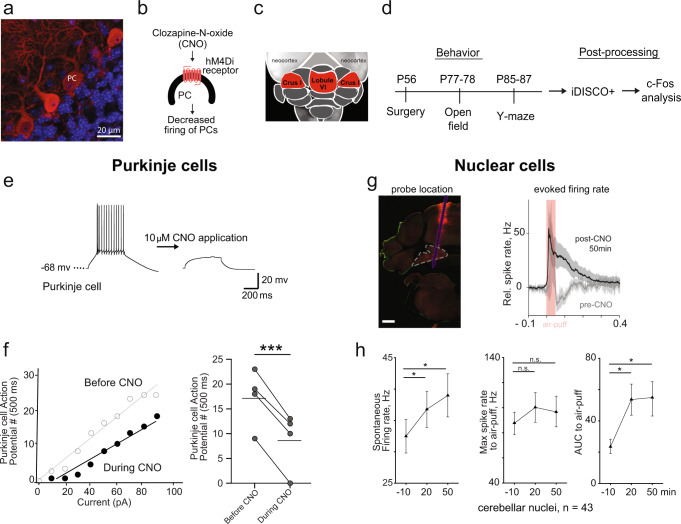
Acute adult inactivation of Purkinje cells in the cerebellar cortex. **a** Expression of the chemogenetic DREADD probe (hM4Di-mCherry) in Purkinje cells (63× magnification, red). **b** The activating ligand CNO binds to the hM4Di receptor and decreases Purkinje cells firing. **c** Dorsal view of the cerebellum with targeted lobule VI or crus I (red). **d** Adult mice received surgery for AAV injection (DREADDs or mCherry) at PND 56 for behavioral testing starting with open field between PND 77-78 and water Y-maze starting between PND 85-87. For each behavior test, animals received CNO (i.p.), vehicle, or no treatment 20 min prior to testing. After all behavior testing ended, brains were taken and cleared for light-sheet microscopy using iDISCO+ and analysis of c-Fos immunopositive cells. **e** Slice electrophysiology of Purkinje cells in five mice before and during application of CNO (10 μM). **f** After activation of DREADDs, Purkinje cells (*n* = 4) fire fewer action potentials in response to current injection. **g** Example histological section with the probe location marked by CM-DiI (red), scale bar, 0.5 mm. Average response (averaged across all sensory airpuff stimulation and all sorted units, background subtracted and normalized) in deep cerebellar nuclei (mean ± 95% confidence interval) before then 20 and 50 min after CNO injection (*n* = 5 mice). **h** Post-CNO injection deep cerebellar nuclei demonstrated increased spontaneous firing rate over 50 min, no change in max spike rate to air-puff, and increased area under the curve (AUC) after an air-puff. Error bars indicate mean ± SEM. Comparisons were made using a paired t-test. *p < 0.05, ***p < 0.001.

To probe the impact of cerebellar activity on flexible behavior and whole-brain activity, we chemogenetically inhibited Purkinje cells. Purkinje cells influence the rest of the brain via their projections to the deep cerebellar nuclei (DCN), which send excitatory output to the rest of the brain. The inhibitory DREADD (Designer Receptor Exclusively Activated by Designer Drugs) hM4Di was expressed in Purkinje cells using an adeno-associated virus (AAV) containing the hM4Di sequence under control of the L7 promoter. DREADD expression was robust and confined to Purkinje cells (Fig. 1a–d and Supplementary Fig. 1).

To trace the influence of DREADD perturbation through the cerebellar circuit, we recorded from Purkinje cells in slices and DCN neurons in vivo. In slices allowed us to easily identify hM4Di-positive Purkinje cells and record from them using whole-cell patch clamp. Application of the DREADD agonist clozapine-N-oxide (CNO; 10 µM) reduced evoked action potential firing (*n* = 4 hM4Di-positive Purkinje cells, paired t-test, *p* = 0.0009) and shifted the input–output curve of firing downward (Fig. 1e-f). To test the consequences of inactivating these neurons, we made many-electrode recordings in five awake mice at the site of Purkinje-cell convergence, the DCN. We identified a total of 43 h-long recorded units in which tactile airpuff stimuli evoked at least a 5 Hz increase in firing (Fig. 1g, h and Supplementary Fig. 2a–d). During these recordings, intraperitoneal injection of CNO increased spontaneous firing rates (−10 min relative to CNO: 32.5 ± 2.7 Hz; 20 min: 36.8 ± 2.9 Hz; 50 min: 39.0 ± 3.4 Hz; mean ± SEM; paired t-test: *p* = 0.033 for −10 vs 20 min, *p* = 0.013 for 20 vs 50 min) (Fig. 1g and Supplementary Fig. 2e, f), consistent with the removal of Purkinje-cell inhibitory input.

Before CNO application, tactile airpuffs first evoked an increase in DCN activity with short latency, consistent with the expected effects of direct mossy-fiber and/or climbing-fiber drive; followed by a strong suppression, consistent with feedforward inhibition of DCN neurons by Purkinje cells. DREADD activation showed a weak tendency to increase the immediate airpuff-evoked increase in firing rate (−10 min: 88.0 ± 8.9, 20 min: 100.4 ± 11.6; 50 min: 97.1 ± 12.2 Hz, mean ± SEM; paired t-test: *p* = 0.061 for −10 vs 20 min, *p* = 0.242 for 20 vs 50 min) with no detectable change in latency (−10 min: 22.3 ± 3.3 ms, 20 min: 26.8 ± 3.0, 50 min: 26.1 ± 3.3 ms from stimulus onset, mean ± SEM; paired t-test: *p* = 0.108 for −10 vs 20 min, *p* = 0.517 for 20 vs 50 min) (Fig. 1h). DREADD activation did abolish the post-stimulus suppression, leaving a sustained increase in firing that outlasted the duration of the airpuff (area under the curve −10 min: 25.2 ± 3.0, 20 min: 56.5 ± 9.7, 50 min: 57.2 ± 11.0 Hz). Taken together, these recordings show that DREADD-induced decreases in Purkinje cell activity lead to increased and prolonged DCN activity.

Posterior vermis (lobule VI and VII) and ansiform area (crus I and crus II) have been implicated in non-motor executive functions^16,34–36^. We stereotaxically injected virus into either lobule VI or crus I (Allen Brain Atlas ansiform area) at postnatal day (PND) 56 and at sacrifice quantified mCherry-positive voxels (Supplementary Fig. 1). Expression arising from injection of these two targets was restricted to the target, with principal spillover into lobule VII and crus II, respectively (Supplementary Fig. 1d). We administered CNO (1 mg/kg intraperitoneal) on test days, which fell between PND 77 and PND 90, and tested animals on two paradigms for assessing flexible behavior: reversal learning in a water Y-maze, and spontaneous behavior in an open-field arena (Table 1). At different points of Y-maze training, we sacrificed mice and collected tissue for whole-brain imaging of the activity-dependent immediate early gene c-Fos (Fig. 1d).

**Table 1:** Experimental groups denoting DREADD and CNO conditions.

Group	DREADDs	CNO	Open Field (N)	Y-maze (N)
Untreated^a^	no	no	60	63 (32 no reversal)
Vehicle only	no	no	9	9
CNO only	no	yes	12	22 (7 no reversal)
mCherry	no	yes	10	18
Lobule VI	yes	yes	10	16 (10 no reversal)
Bilateral Crus I	yes	yes	10	7
Crus I right	yes	yes	10	25
Crus I left	yes	yes	10	26
L7Cre; Tsc1^flox/flox*^	no	no	9	0

Animals underwent surgery for an injection of DREADD or mCherry AAV expression. *N* denotes the number of mice used for each type of experiment. Prior to open field and the water Y-maze task, animals were injected with the DREADD ligand (CNO), Vehicle, or remained untreated.

^a^Includes some data initially gathered for ref. ^50^.

### Cerebellar disruption of lobule VI or bilateral crus I impairs Y-maze reversal learning

**Fig. 2:**
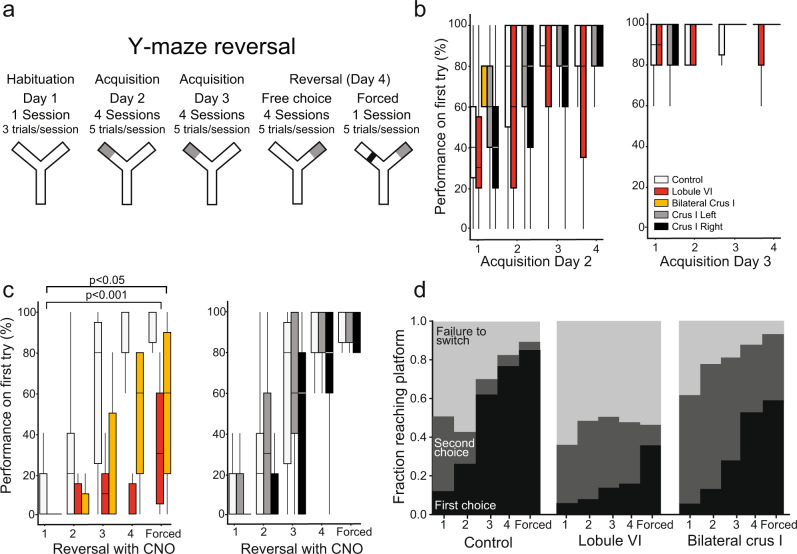
Effect of lobule VI and crus I inactivation on behavior. **a** Protocol for water Y-maze reversal consisting of habituation, 2 days of training (day 2 and day 3), and one reversal day ending in a forced session (day 4). **b** Animals learned to find the platform over 2 days. First try performance of 80% at the end of session 4 was required to continue to reversal. **c** Y-maze reversal was impaired in animals with lobule VI (*n* = 16) and bilateral crus I (*n* = 7) inactivation compared to CNO only control (*n* = 22). Unilateral effects were not found compared to controls (crus I right *n* = 25, crus I left *n* = 26). *P* values indicate between-group comparisons of the entire time series by repeated-measures ANOVA. **d** Typical mice learned through trial and error, demonstrating more accurate performance over time. Lobule VI and crus I perturbed mice stayed repeatedly with the original incorrect choice compared to CNO only control. Significance values in (**c**) indicate comparisons using a generalized mixed effect model (GLMM) with a binomial distribution. All box-and-whisker plots show quartiles of the data and whiskers extend to show distribution. *p < 0.05, **p < 0.01.

To test flexible learning, we trained mice in a water Y-maze (Fig. 2). After 1 day of habituation to the environment, an underwater platform was placed at the end of one Y-arm and the mice spent two days learning to find the platform through trial and error (acquisition days 2 and 3). On day 4, the platform was switched to the opposite arm for four sessions (reversal). On the fifth and final session on day 4, a barrier was placed blocking the originally learned side (forced reversal) (Fig. 2a). On all days, we defined the correct choice as an entrance to the correct arm and climbing onto the platform during a 40-s trial.

All DREADD-activated groups showed a similar time course of initial acquisition, showing no statistically detectable differences compared with controls (generalized linear mixed-effect model, GLMM, *p* = 0.76; Fig. 2b; for control experimental design, see “Methods”). Bilateral inactivation of crus I showed a tendency to increase performance on initial training (Fig. 2b), reminiscent of our recent observation that crus I activation leads to accelerated acquisition on an evidence accumulation task^37^. Control treatments did not affect distance swum, initial learning, or reversal learning compared with untreated mice (Supplementary Table 1, Supplementary Fig. 3a–d).

Lobule VI-perturbed mice and bilateral crus-I-perturbed mice were strongly impaired in reversal learning (Fig. 2c and Supplementary Fig. 3e); lobule VI compared to CNO-without-DREADD controls (CNO only reversal) (comparison of entire time series by repeated measures ANOVA, *p* = 8.9 * 10^−6^, entire time series Cohen’s *d* = 3.84 and bilateral crus I compared to control *p* = 0.04, Cohen’s *d* = 1.93). Performance on the forced-reversal session was also reduced (lobule VI, *p* = 0.0039) (Supplementary Movie 1). In tests for lateralization of crus I function^38^, we found that perturbation by crus I injection on either left (*p* = 0.83) or right (*p* = 0.10) side alone was insufficient to impair reversal significantly (Fig. 2c).

To probe behavioral patterns of this learning failure, we analyzed individual trials. Entrance into any arm N times, ending with a landing in the correct arm, was defined as an N-th choice trial. Even in the first reversal session, control mice (CNO only) typically found the platform in their first or second choice (85% of mice), eventually making a correct first choice in 71% of trials by the fourth session. In contrast, lobule-VI-perturbed mice made persistent errors even by the fourth session, making correct first choices on 16% of trials and correct second choices on 34% of trials (Fig. 2d and Supplementary Fig. 3f). In forced-reversal trials, lobule-VI-perturbed mice displayed a unique perseverative behavior of swimming back and forth between the divider and beginning of the maze instead of switching to the obvious open arm, resulting in animals failing to switch in 64% of forced-reversal trials (Supplementary Movie 1). We also observed perseveration in crus-I perturbed mice, whereby mice continued to swim to the previously correct arm and failed to switch in 47% of forced-reversal trials. (Fig. 2d and Supplementary Fig. 3f). In summary, mice typically learned the Y-maze by trying multiple arms until they found the platform, but perturbing the cerebellum resulted in persistent errors and a failure to reverse.

### Whole-brain c-Fos reveals lobule-specific targets of influence

**Fig. 3:**
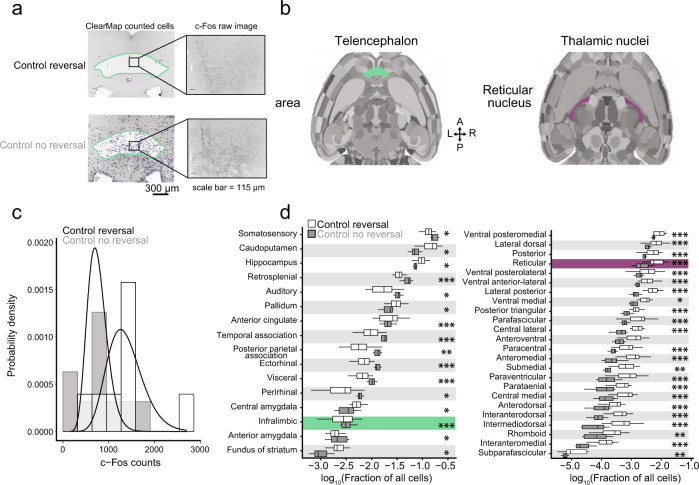
Whole-brain analysis of c-Fos in CNO only controls. **a** Example of ClearMap cell counting in the infralimbic area in CNO only reversal (*n* = 22) compared to CNO only no reversal (*n* = 7). **b** Example horizontal mouse brain sections representing infralimbic area (green) and reticular nucleus (purple). **c** Negative binomial regression under two CNO only controls undergoing reversal or no reversal. This example is the infralimbic area. Nonparametric kernel density estimates derived from count-based densities (bars). Solid curves indicate the probabilities at each count level from a fitted negative binomial regression model. **d** Fraction of all cells (log_10_) in the telencephalon and thalamus comparing CNO only reversal (white) and no reversal (gray). Comparisons were made using a negative binomial regression. *p < 0.05, **p < 0.01, ***p < 0.001.

**Fig. 4:**
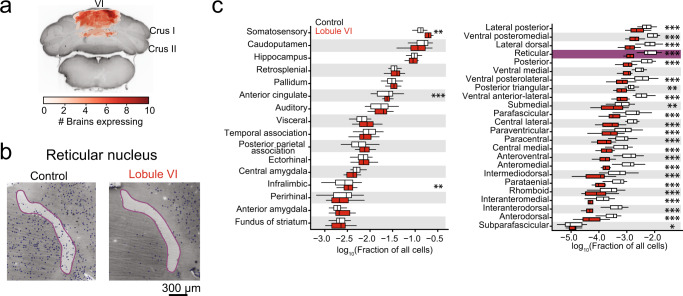
Effects of lobule VI perturbation on whole-brain c-Fos. **a** Overlay of regions of DREADD-AAV expression recovered using immunofluorescent labeling of mCherry and lightsheet microscopy imaging. **b** Example images of ClearMap cell counting in the reticular nucleus comparing Lobule VI (*n* = 16) and CNO only control after reversal (*n* = 22). **c** Fraction of all cells (log_10_) by total regions analyzed in the telencephalon and thalamus comparing Lobule VI and CNO only after reversal. Comparisons were made using a negative binomial regression. Yellow: *p < 0.05, Green: **p < 0.01, Red: ***p < 0.001.

**Fig. 5:**
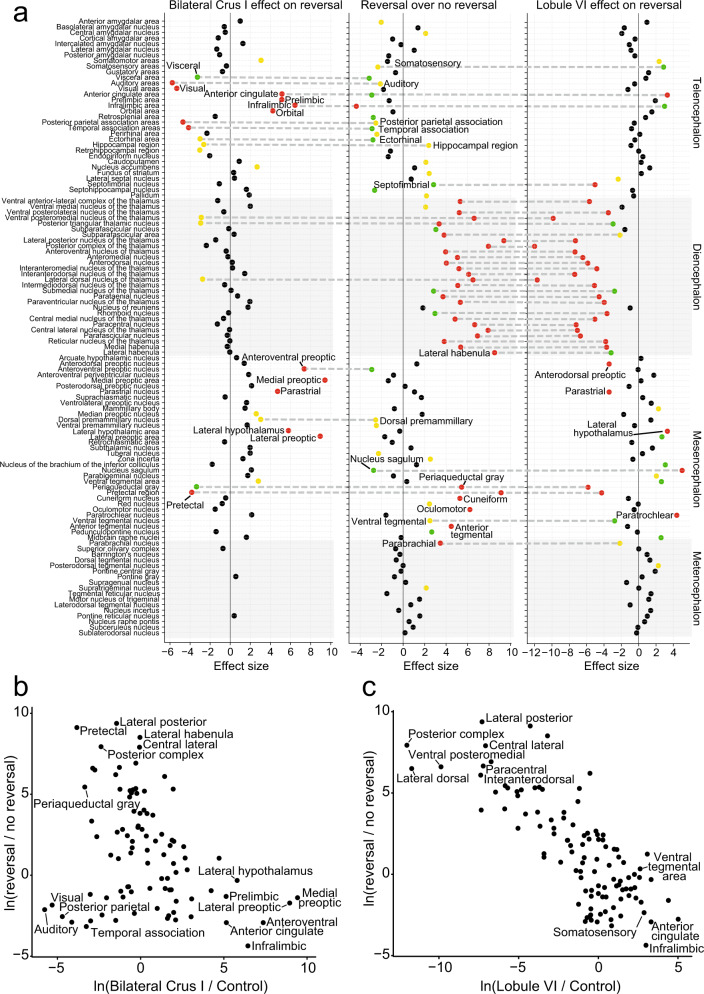
Brain-wide association study (BWAS) to identify activated regions of c-Fos expression. **a** Statistically-significant (*p* < 0.05) bilateral crus I (*n* = 7), reversal (*n* = 22), and lobule VI (*n* = 16) structures compared to CNO only controls. Lobule VI perturbation modulated regions necessary for reversal learning, including thalamus and anterior cingulate cortex. Crus I bilateral perturbation altered regions in the telencephalon, including prelimbic, infralimbic, anterior cingulate, and orbital frontal cortex. **b** Correlations between bilateral crus I (*n* = 7) and reversal learning structures (Pearson’s *r* = −0.29). **c** A strong negative correlation was found between lobule VI and reversal learning structures (Pearson’s *r* = −0.78). All data were calculated as the natural log of ratios rescaled by the standard error. Comparisons were made using a negative binomial regression. Bilateral Crus I and lobule VI were compared to CNO only controls. Yellow: *p* < 0.05, Green: *p* < 0.01, Red: *p* < 0.001.

We next sought to identify brain-wide targets of cerebellar influence that could account for the observed inflexible behavior. To quantify expression of the transiently-expressed, activity-dependent immediate early gene c-Fos^39,40^, we extracted brains at different points in the Y-maze learning and reversal paradigm^39^. We compared the effects of lobule VI-targeted and crus I-targeted perturbations with subtractive control conditions closely matched for activity levels and previous learning (see Methods for description). We then cleared the brains using iDISCO+^41^ and immunostained them for c-Fos and the mCherry fluorescent tag encoded by both DREADD and control AAVs (Supplementary Fig. 4a, Supplementary Movie 2, Supplementary Table 2 for reagents). Samples were imaged for AlexaFluor-647 on a light-sheet microscope, aligned to the Princeton Mouse Atlas^1–5,42^, analyzed for c-Fos positive cells using ClearMap, and validated ClearMap using human annotators (Fig. 3a, Supplementary Fig. 4c and Supplementary Movie 3)^43^. Finally, using 122 chosen structures, we created a 3-D representation of the data for each brain using Neuroglancer, a Google WebGL-based viewer for volumetric data (Fig. 3a and Supplementary Fig. 5) for analysis of c-Fos in single or combined regions. Our various paired experimental comparisons are described in detail in Supplementary Table 3. In each comparison, we processed brains from all control and treatment animals as a single batch using the same tissue preparation and imaging conditions whenever possible. In the few cases where we needed multiple batches, we adjusted for confounding batch effects by including indicator variables for batches as covariates in regression models. Analogous to genome-wide association studies, we analyzed each brain region independently and corrected the results post hoc for false discovery rate. For each region, we quantified the contrast between counts for the animals in the treatment group versus the control group using negative binomial regression with a log link function. To account for animal-specific variation in total count, we included the log of total counts as an offset (Fig. 3c). To identify brain regions activated in the initial acquisition of Y-maze learning, we assayed c-Fos-positive cells immediately after 3 days of Y-maze ending on the last initial acquisition day (*n* = 10 mice), using for baseline comparison animals that underwent habituation-only on the first day of Y-maze (*n* = 10 mice; Supplementary Fig. 6). Out of 122 regions, 33 regions showed increased activity and 6 regions showed decreased activity. In the cerebellum itself, we found c-Fos signal in very few scattered cells, consistent with previous observations that activation of Purkinje cells leads to delayed expression principally in cytoplasm^44^. We found activation in thalamus (1.4-fold) and in prelimbic (1.8-fold) and temporal association (1.65-fold) cortex. In the rest of the brain, we found some of the strongest associations in parabrachial nucleus (2.8-fold), basolateral, central, and cortical amygdalar nucleus (1.8, 1.7, and 1.7-fold), lateral habenula (2.3-fold), periaqueductal gray (2.1-fold), septohippocampal nucleus (2.9-fold), and lateral septal nucleus (2.6-fold) (Supplementary Fig. 7a, b). These increases occurred despite a 57% decrease in distance swum (first day, 54.6 ± 4.4 cm; third day, 23.4 ± 6.2 cm, mean ± SD) (Supplementary Table 1). We confirmed results in several of these regions using conventional immunohistochemistry (parabrachial nucleus: *p* = 0.00006, Cohen’s *d* = 1.4; thalamus:*p* = 0.000002, Cohen’s *d* = 2.3) (Supplementary Fig. 8a–c). Overall, initial learning specifically activated a wide range of regions linked with associative and affective function. We analyzed specific neural correlates of reversal learning by performing a subtractive comparison between two closely related conditions: day 4 after one day of reversal learning (*n* = 10 mice), and day 3 of acquisition training alone, i.e. the immediately preceding day (*n* = 8) (Fig. 3). The distance swum between the conditions differed modestly by a ratio of 1.2, comparable to the difference in time swimming, 200 vs. 160 s (Supplementary Fig. 9 and Supplementary Table 1; Cohen’s *d* = 0.3). Reversal learning resulted in 16 decreased and 44 increased brain structures compared to the third day of acquisition. We found statistically significant activation throughout thalamus (2.7-fold), including polymodal regions (3.2-fold) as classified by ref. ^45^, sensory/motor regions (2.1-fold), and the reticular nucleus, which is modulatory (3.2-fold)^45,46^. Reversal learning was associated with decreases in c-Fos cell counts in the majority of neocortical regions, the largest change being a decrease in infralimbic cortex activity (0.56-fold, Fig. 3b, d). Additional activation occurred in medial and lateral habenula (15.6-fold and 7.6-fold), periaqueductal gray (1.9-fold), and parabrachial nucleus (1.6-fold) (Fig. 3b–d, Supplementary Fig. 5, Supplementary Fig. 7a, b). Fig. 4 shows the activation patterns for reversal with and without lobule VI perturbation. We tested the effects of lobule VI-targeted perturbation (*n* = 10 mice) on c-Fos generation in reversal learning. Perturbation of lobule VI (Fig. 4a) during reversal learning resulted in a reduction in c-Fos activity (Fig. 4b) throughout the thalamus (overall 0.28-fold compared with unperturbed reversal learning). Lobule VI control (DREADD surgery plus CNO under a third day of acquisition) did not result in similar patterns of c-Fos, suggesting lobule VI perturbation uniquely alters patterns of c-Fos activation in reversal learning (Supplementary Fig. 10). The distance swum between lobule-VI perturbation and unperturbed reversal differed by a factor of 1.1 (Supplementary Fig. 9 and Supplementary Table 1). By region, brain structures showed statistically significant differences in activity as follows: Increased activity was seen in somatomotor, somatosensory, anterior cingulate, and infralimbic cortex. Midbrain regions both increased (ventral tegmental area, lateral hypothalamus, midbrain raphe nuclei) and decreased (parastrial, medial and lateral habenula, and periaqueductal gray) in activity (Fig. 4c, Fig. 5a, c, Supplementary Fig. 8d–f, and Supplementary Fig. 7a, b). The overall pattern of changes is shown in Fig. 5a. To compare this pattern of differences with the pattern arising from other perturbations, Fig. 5 shows differences arising from targeted perturbations of specific lobules with AAV-DREADD injection, in all cases during reversal learning, with CNO-without-DREADD as the baseline condition. For bilateral crus I-targeted perturbation (*n* = 7), the distance swum between bilateral crus I-targeted perturbation and reversal learning differed by a ratio of 1.3, with bilateral crus I-targeted swimming more (Supplementary Fig. 9 and Supplementary Table 1). By region, brain structures showed the following changes in activity: Bilateral perturbation of crus I during reversal learning did not lead to statistically detectable differences in thalamic activity compared with unperturbed reversal learning. However, differences were seen in the neocortex, both increased (anterior cingulate, prelimbic, infralimbic, and orbital) and decreased (auditory, visual, posterior parietal, and temporal) activity. Increases were also seen in parastrial nucleus and hypothalamus (lateral and preoptic) (Fig. 5a, b). Statistically significant changes were not seen after unilateral perturbation of crus I (crus I right *n* = 25, crus I left *n* = 26) (Supplementary Fig. 6).

Many effects of lobule VI and crus I perturbation went in the opposite direction as the reversal-versus-acquisition condition. Of the 61 regions showing changed activity in reversal learning, opposite-direction changes occurred in 47 regions from lobule VI perturbation, and in 27 regions from crus I bilateral perturbation. Opposite-direction changes encompassed the majority of thalamic regions and lateral and medial habenula, as well as selected regions in telencephalon (anterior cingulate, infralimbic) and mesencephalon (periaqueductal gray, pretectal regions) with a lobule VI perturbation (Fig. 5a). The overall pattern of cell ratios was strongly correlated with the reversal-versus-acquisition condition for lobule VI (correlation of log-cell-ratios by Pearson’s *r* = −0.78) (Fig. 5c) and less correlated for bilateral crus I (Pearson’s *r* = −0.29) (Fig. 5b), and spanned a similar range of values in both cases. Thus, lobule VI perturbation reversed activation patterns in most regions that were activated by reversal learning, while crus I perturbation had effects that were limited to neocortical and hypothalamic regions.

Initially upon chemogenetic perturbation of lobule VI or crus I, activity is likely to change in trisynaptically connected neocortical targets. To test the extent to which this primary connectivity constrained the observed changes in c-Fos signal, we compared the rank order of changes in these targets with the rank order of connectivity as determined by anterograde transsynaptic tracing from the cerebellar cortex^5^. The Spearman correlation between c-Fos signal and anatomical connection strength to neocortex was for lobule VI +0.62 (*p* = 0.10) and for crus I disruption, the correlation was +0.74 (*p* = 0.04) (Supplementary Fig. 11). This correlation indicates that patterns of activation may be at least partly explained by connectivity.

### Within each experimental group, reversal learning activates functional networks that are disrupted by cerebellar perturbation

**Fig. 6:**
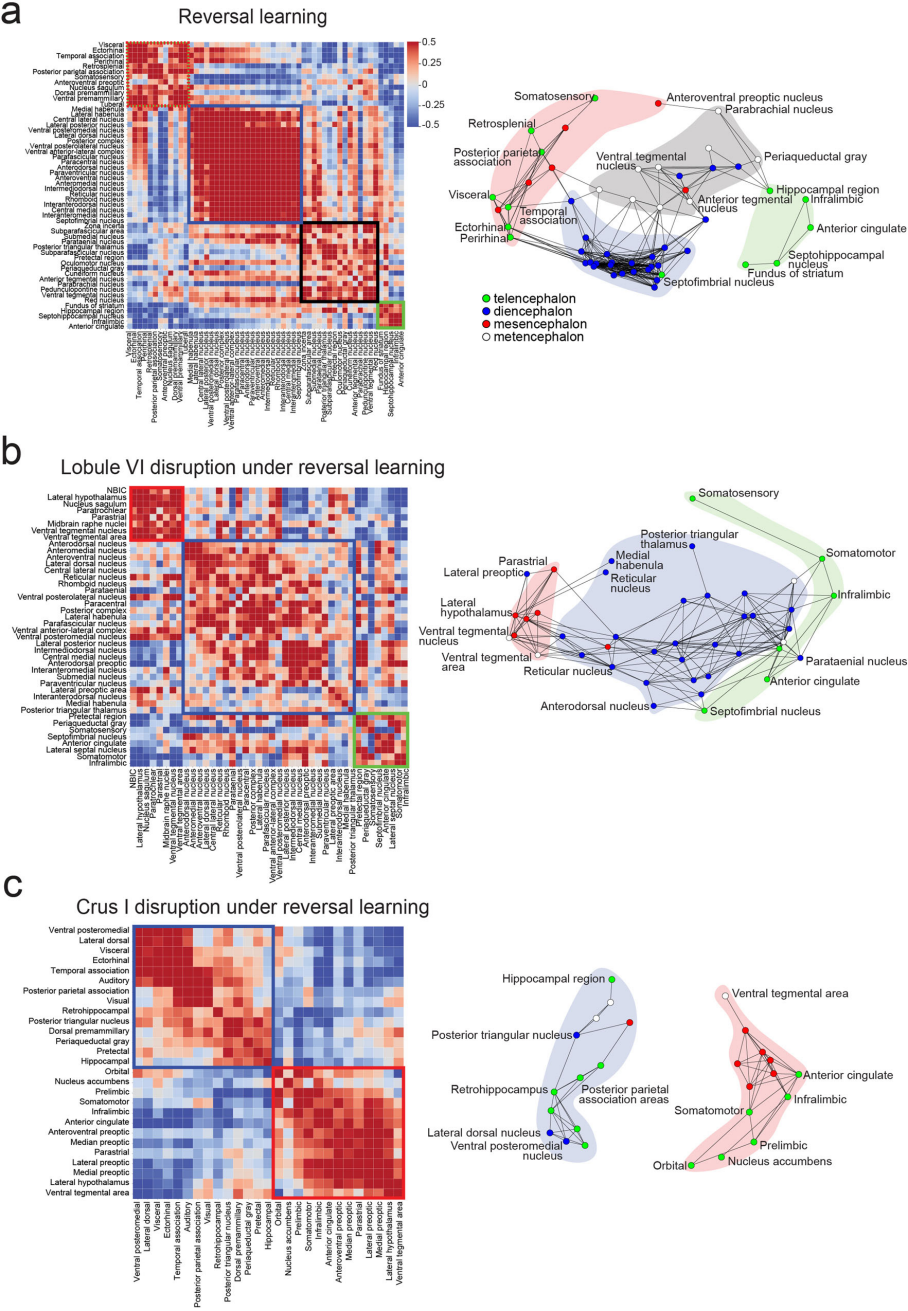
Cerebellar influence on Y-maze reversal networks. Correlation matrices of all brain regions within (**a**) reversal learning (CNO only reversal), (**b**) lobule VI disruption, (**c**) crus I disruption (all crus groups combined). Correlation matrices of significant brain regions (*p* < crus I 0.05) showing inter-region connections for c-Fos expression with (**a**) the reversal learning group (CNO only reversal), (**b**) lobule VI disruption, and (**c**) crus I disruption (all crus groups combined). Strength of correlation reflected in scale bar (Spearman’s rho). Network graphs were generated to visualize relationships between major brain structures (mesencephalon: red, telencephalon: green, metencephalon: white, diencephalon: blue) based on correlations in (**a**) CNO only reversal (*n* = 22 mice, 54 regions, rho ≥ 0.46, FDR = 0.05), (**b**) lobule VI disruption (*n* = 16 mice, 42 regions, rho ≥ 0.55, FDR = 0.05; and (**c**) crus I disruption (*n* = 58 mice, 27 regions, rho ≥ 0.40, FDR = 0.0025). Lobule VI and crus I inhibition both reduce thalamic connectivity that occurs under Y-maze reversal learning. NBIC Nucleus of the brachium of the inferior colliculus.

To examine functional connectivity arising during reversal learning, we calculated the Spearman rank coefficient ρ between pairs of brain regions among the mice within any one c-Fos experimental group (Fig. 6, Supplementary Figs. 12–18)^47,48^. Under the reversal learning condition (Fig. 6a and Supplementary Fig. 12, *n* = 22 animals), thalamic regions showed strong and widespread correlations with one another, consistent with the idea that the strong activation of thalamus in reversal learning (Fig. 5a) is driven by a shared source of regulation that affects many regions in concert. Within-group reversal learning (*n* = 22 animals) also showed both positive and negative correlations between pairs of neocortical regions. The lobule VI group (*n* = 16 animals) showed similar but weaker patterns of correlation in thalamus (Fig. 6b and Supplementary Fig. 13), suggesting that lobule VI perturbation removes a shared source of drive common to these regions.

For crus I (Fig. 6c, Supplementary Fig. 14 and 17), to attain a large sample size we combined the bilateral inhibition group (7 animals) with the left (26 animals) and right (25 animals) inhibition groups, which have the same synaptic targets and whose effects on behavior were smaller and statistically harder to detect (Fig. 2c, Supplementary Fig. 17a). Analysis of c-Fos in the left and right groups (Supplementary Fig. 17c, d) separately gave similar results as the bilateral group (Supplementary Fig. 17b). Compared with the unperturbed group, both lobule VI and crus I perturbation conditions had stronger within-group correlations in mesencephalon and metencephalon (Fig. 6).

To identify functional networks of likely importance, we restricted analysis to structures found to be significant in brainwide comparisons of groups at *p* < 0.05 in Fig. 5: for reversal learning, compared with initial acquisition, and for lobule VI and crus I groups, compared with unperturbed reversal learning (Fig. 6 and Supplementary Figs. 13–16). To visualize the overall pattern of correlation, we used multidimensional scaling to reduce the matrix of correlations to points in a plane, where each point represents one brain region. The more correlated the activity between two regions, the closer the points appear in the plot (Fig. 6). Points were joined by line segments if the absolute value of the correlation exceeded a threshold of 0.4, or a higher value as necessary to lower the false discovery rate to 0.05^49^.

In reversal learning, we found multiple groups of regions within which correlations exceeded threshold (Fig. 6a). Diencephalic regions, which encompassed thalamic nuclei, lateral habenula, and paraventricular nucleus, showed the strongest internal correlations. This activity was generally anticorrelated with activity in neocortical regions. Neocortical regions were separated into two groups that were not joined to one another by strong pairwise correlations. One group included ectorhinal, and retrosplenial cortex, and the other group included anterior cingulate and infralimbic cortex.

Inhibition of lobule VI reduced the degree of correlation among thalamic nuclei, and showed strong correlations within mesencephalon, and weak correlations within neocortex (Fig. 6b). Such a pattern might be expected if lobule VI exerted influence via an intermediate source of global thalamic regulation, such as the reticular nucleus. In contrast, within the all-crus-I group, correlations comprised largely mesencephalic and telencephalic regions. Within-neocortex correlations formed two groups with no mutual correlations: one including frontal regions (prelimbic, orbital, infralimbic and somatomotor areas, as well as nucleus accumbens) and another for spatial orienting and memory (ectorhinal area, temporal association area, and hippocampal regions) (Fig. 6). Such a pattern might be expected if crus I influence over neocortex pass through multiple pathways in thalamus and other midbrain structures. These patterns were not seen in vehicle-control-treated mice (*n* = 9 mice; Supplementary Fig. 18). In summary, we found that regions specifically activated during Y-maze reversal learning formed thalamocortical functional networks that were disrupted in different patterns after perturbation of lobule VI and crus I.

We tested whether the amount of c-Fos-expression in a region influenced the ability of correlation analysis to identify connected nodes. To do so, we pooled all experimental animals into a single group of 169 mice, and then divided brain regions into groups of low (28 regions), medium (27 regions), and high (28 regions) c-Fos cell counts (fraction of all cells). We found that medium-cell-count regions had a higher proportion of connected pairs (46 of 325 pairs, or 14.1%) than low-expressing regions (22 of 351 pairs, or 6.3%; Fisher exact probability test, *p* = 0.0008), and also, to a modest degree, high-expressing regions (31 of 351 pairs, or 8.8%; Fisher exact probability test, *p* = 0.04). These findings indicate that inter-region correlations show a potential tendency to be less frequent or harder to detect in regions with high cell counts.

### Lobule VI and crus I modulate multiday behavioral habituation to an open field

**Fig. 7:**
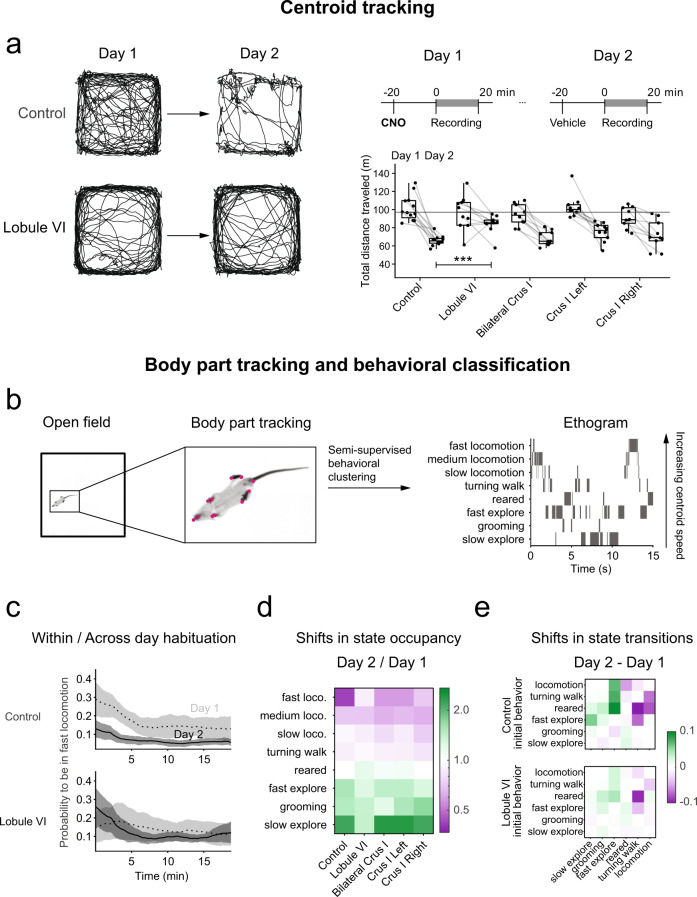
Effects on spontaneous behavior after perturbation of lobule VI and crus I. **a** Lobule VI-perturbed (*n* = 10) mice show a larger total distance traveled on the second day compared to a day-matched CNO only control group (*n* = 12). Example trajectories of the centroid position of a lobule VI-perturbed mouse and a control mouse in the open field for the first 5 min. Timeline of open-field experiments. Comparisons were made using an one-way or two-way mixed ANOVA to compare total distance traveled between day 1 and 2 of control (CNO only), Lobule VI, bilateral crus I (*n* = 10), crus I left (*n* = 10), crus I right (*n* = 10). **b** Machine learning pipeline to obtain ethograms for the open-field recordings. First, body parts were tracked using LEAP. These postures were processed, as described in ref. ^50^. to assign one of six behaviors to each time point in the recording. Locomotion was divided into slow, medium and fast based on the centroid velocity of the mouse. **c** The probability to be in the fast locomotion state decreased within and across days for the CNO only control group, but lobule VI-perturbed mice show a higher or similar probability to be in fast locomotion at the beginning of the experiment on the second day compared to the first day. **d** The state occupancies for lobule VI-perturbed mice changed less across days than the other groups, indicating a lack of behavioral habituation. **e** The state transitions for lobule VI-perturbed mice changed less across days than control group, indicating a lack of behavioral habituation. ***p < 0.001.

To test whether the consequences of cerebellar perturbation extended to more complex behavior, we measured the capacity of mice to adapt behaviorally to a novel environment. We characterized spontaneous behavior in an open-field arena^50^ recording mouse behavior by imaging from beneath for 20 min over two days in order to track location and to allow automated tracking of body parts using the LEAP (LEAP Estimates Animal Pose) software package^51^, a neural network trained to track the positions of 18 joints (Fig. 7a, b and Supplementary Movie 4).

We recorded animals for 20 min on each of two days, after a dose of CNO on day 1 and a dose of vehicle on day 2. Over successive days, control mice that did not receive AAV reduced their daily amount of locomotion (two-way mixed ANOVA *F*(1,47) = 146, *p* < 0.001). Disruption of lobule VI activity prevented this behavioral habituation. Lobule VI-perturbed mice traveled significantly more on the second day (*d* = 2.3, one-way ANOVA F(4,47) = 5.04, *p* = 0.002, Dunnett post-hoc test *p* < 0.001) compared to the control group. The total distance traveled on day 2 was not significantly different from the distance on day 1 in lobule VI perturbed mice, in contrast to a decrease for mice with a unilateral or bilateral disruption of crus I (*d* = 2.6, 2.4, and 1.4 for bilateral crus I, left crus I, and right crus I), or control mice (*d* = 3.4, repeated-measures ANOVA for each group, *p* < 0.01, Bonferroni correction) (Fig. 7a).

Perturbation of lobule VI or bilateral crus I did not change the fraction of time mice spent in the inner region of the open field arena compared to control animals on day 1. Right crus I-perturbed animals did spend a significantly larger fraction of time in the inner region of the open field arena on day 1 (*r* = 0.6, Kruskal-Wallis test chi-squared(4) = 9.52, *p* = 0.049, pairwise comparisons using Wilcoxon rank sum exact test *p* = 0.02, Benjamini-Hochberg correction) (Supplementary Fig. 19a). None of the manipulations affected locomotory gait (Supplementary Fig. 19b, c) or spatial preferences in the arena such as locomotion in the periphery and grooming in corners (Supplementary Fig. 19d).

To explore the structure of this altered behavior, we used automated pose analysis. We performed semi-supervised behavioral clustering on LEAP-tracked body-part locations to identify six clusters of body dynamics: slow exploration, grooming, fast exploration, rearing, turning walk, and locomotion. We took the clusters as behavioral states to generate an ethogram, arranged in order of increasing centroid speed (Fig. 7b). Since mice spent the most time in locomotion, we further subdivided locomotion into three groups, slow, medium, and fast, based on centroid velocity (Supplementary Fig. 19c).

The fractions of time spent in each of the eight behaviors were significantly different for lobule VI-perturbed mice compared to the control animals. More specifically, compared with control animals, lobule VI disruption led to decreases in time spent in the rearing state on day 1 and increases in fast locomotion on day 2 (Fig. 7c, d and Supplementary Fig. 20a,b, d). The ratio of fast locomotion in lobule VI-perturbed mice compared to the control group on day 2 was 1.7, sufficient to account for the increased total distance traveled on day 2 compared to the other groups. Within each day’s 20 min of observation, the probability of being in the fast locomotion state decayed over time. However, lobule VI-targeted, bilateral crus I-targeted, and right crus I-targeted mice were more likely to perform fast locomotion just after the experiment started on day 2 compared to day 1, in contrast to our observations for the control group and left crus I perturbation (Fig. 7c and Supplementary Fig. 20b).

We calculated complex behavioral habituation as the ratio of time spent in each state for day 2 compared to day 1. There were few differences among the various controls, except that compared with vehicle-only mice, CNO-exposed animals had a slight reduction in locomotion and more behavioral habituation (ratio further from 1) for fast locomotion and turning walk (Supplementary Fig. 20c, d). Overall, the behavioral habituation ratio was closer to 1 for lobule VI-targeted mice for most behavioral states, especially fast locomotion and slow exploration (Fig. 7d and Supplementary Fig. 20c). We next examined transition probabilities between behavioral states. In control animals, day 2 probabilities compared to day 1 showed higher transition frequencies in the direction of less-active states (i.e. above the diagonal of the matrix in Fig. 7e and Supplementary Fig. 19e). This tendency was markedly reduced in lobule VI-targeted animals. In crus-I targeted mice, the same semi-supervised behavioral clustering analysis found subtle differences, in particular a right crus I-induced shift from fast-locomotion state to the slow-locomotion state starting on day 1 (Fig. 7d and Supplementary Fig. 20c). In summary, lobule VI-perturbed animals maintained similar within-day response patterns to the same environment despite impaired behavioral habituation over two days of exposure.

## Discussion

We found that intact cerebellar function in lobule VI and crus I was necessary for two acquired flexible behaviors, choice reversal in a swimming Y-maze and habituation to an open field. In both behavioral paradigms, silencing of Purkinje cells led to deficits that became apparent over a period of several days, and in the case of Y-maze was marked by incorrect repetitive choices even in a forced session. Y-maze choice reversal recruited activity in a diencephalon-centered group of regions, most of which were reversed by inhibition of Purkinje cells in lobule VI, and in prefrontal and hypothalamic regions, which were reversed by inhibition of either lobule VI or crus I. Taken together, these studies comprise a demonstration of cerebellar perturbation leading to specific alterations in whole-brain activity and nonmotor function.

Identification of effects on flexible behavior required us to distinguish them from changes in the coordination of movement. In the case of Y-maze reversal learning, we did this using distance swum, a traditional measure of movement. In the arena, pose analysis^50^ allowed additional detailed analysis in which we could simultaneously analyze the detailed kinematics of limb movement and longer time-scale features of behavior. Such an analysis required a method that could track individual body parts, such as LEAP^51,52^ or other approaches^53–56^. We did not find differences in gait or in spatial occupancy of the arena, suggesting that the chosen cerebellar perturbations affected the evolution of motor behaviors over several days, but not the capacity to interact with the physical environment or generate locomotor behavior. These results are consistent with past work in which rodent gait was not altered by lobule-specific perturbation of posterior cerebellum^16,18^, but was changed by cerebellum-wide disruption^50,57,58^.

Flexible cognition, as examined by Y-maze reversal learning, was found to be strongly modulated by lobule VI and crus I. Mice demonstrated perseveration in this task by swimming repeatedly toward the previously learned arm before finding the platform in the third arm of the Y-maze, even when the incorrect arm was blocked. Perseverative behavior is a principal criterion for autism spectrum disorder^59–61^. Vermal lobules VI–VII are altered in their volume developmental trajectory in ASD children compared with the rest of cerebellum^14,15^, suggesting a specific role for lobule VI in driving ASD-like outcomes. In the open field, lobule VI-perturbed mice demonstrated reduced behavioral habituation by staying in a fast locomotive state on both days. Given the widespread disruption of thalamic activity caused by inhibiting lobule VI output in mice, these behavioral deficits may arise from the disruption of sensory or polymodal processing, reducing the capacity of the forebrain to detect novelty or process its consequences.

We found that disruption of crus I altered the ability to reverse learning in a swimming Y-maze. Human crus I is engaged in the processing of sensory novelty^17,62,63^. Crus I in rats and mice is putatively homologous to human crus I/II^17^ which indicates that our perturbations span the homolog of human ansiform area. Our findings are in accordance with lobule-specific substrates for deficits that arise in cerebellar cognitive-affective disorder^6,7,64^.

The cerebellum influences the rest of the brain through polysynaptic paths through the deep and vestibular nuclei. By analyzing the immediate-early gene c-Fos, we found that reversal learning engaged midbrain, diencephalic, and neocortical regions associated with flexible behavior, and that this engagement was altered by perturbation of lobule VI or crus I. Many of these brain regions receive disynaptic paths from lobule VI and crus I, as demonstrated by transsynaptic tracing^1–5^. In the case of lobule VI, we observed changes in thalamic activity that were consistent with both a broad effect and with specific anatomical paths of connectivity, suggesting that lobule VI might act via thalamic intermediates, including both relay nuclei and nuclei with broad modulatory effects. One candidate for modulatory effect is the reticular nucleus, which receives direct paths from lobule VI^5^. In the case of crus I, effects were strongest in neocortex, where functional networks were altered, suggesting the possibility that crus I may influence corticocortical processing.

In the mesencephalon, the habenula, periaqueductal gray, and parabrachial nucleus are engaged during defensive, negative-reward, and decision-making behavior^65–68^. One of these regions, the parabrachial nucleus, is a major monosynaptic target of Purkinje cells^24^. Finally, the infralimbic and anterior cingulate cortex are activated in effective decision-making and reward-seeking^69–71^. Our observation that lobule VI and crus I inhibition affects activity in these regions during reversal learning suggests that cerebellar activity is necessary for the normal expression of a wide range of brain activity in the face of changing environmental valence.

Recent studies in mice show that vermal and hemispheric regions project via the deep cerebellar nuclei to distinctive patterns of forebrain structures^1–4,72,73^, influencing thalamocortical nonmotor processing. Chemogenetic inhibition of lobule VI Purkinje cells led to broad decreases in thalamic activity as measured using c-Fos. Inhibition of lobule VI also led to a strong loss of functional network correlation among thalamic structures, as well as among neocortical regions. A major target of cerebellar output, especially from lobule VI/VII, is the thalamic reticular nucleus^1–5^, which is inhibitory and sends its outputs throughout the rest of the thalamus. Reticular nucleus paths have been suggested to have a gating effect on thalamocortical function^74^ and are important for flexible behavior^75–78^. In addition, removal of Purkinje-cell inhibition in lobule VI might be expected to increase activity in thalamic polymodal nuclei via cerebellothalamic excitation. The fastigial nucleus, which receives strong lobule VI input, has recently been shown to send output to brainstem targets subserving arousal and autonomic functions^73^. The widespread nature of lobule VI’s functional targets supports the idea that the cerebellum acts as a powerful modulator of thalamocortical processing during flexible behavior. Our findings suggest that lobule VI pathways are also necessary for coordination among thalamic regions.

Chemogenetic inhibition of crus I Purkinje cells also led to changes in neocortical activity as measured using c-Fos. Human and rodent crus I^1,2,4,5,79^ project to thalamus, both sensory/motor such as ventral posteromedial nucleus and polymodal such as lateral dorsal nucleus, and to hypothalamus, which like the thalamus is a diencephalic structure that projects monosynaptically to neocortex^80^. Transsynaptic paths from rodent crus I project particularly densely to infralimbic, prelimbic, and orbital cortex, providing a substrate for our observed alterations in c-Fos expression^5^. We found that under crus I inhibition, activity in neocortical regions differed strongly from unperturbed reversal learning, and functional network analysis revealed separation of activity into highly distinct sensory and associative neocortical networks, suggesting that during flexible Y-maze learning, crus I plays a role in coordinating the action of these two networks.

As a marker of targets of cerebellar perturbation, the use of c-Fos carries several limitations. Our c-Fos results could have potentially arisen from either direct and immediate consequences of cerebellar perturbations, or indirect long-term consequences arising after failure to complete the task. Two lines of evidence suggest that at least some effects were direct consequences. First, an indirect consequence such as stress should lead to a similar pattern of change, irrespective of the original perturbation^40^. Lobule VI perturbation changed thalamic c-Fos activation more than it changed neocortex, and crus I perturbation changed neocortex but not thalamus. This difference is consistent with the observation that neocortex receives inputs from many nonthalamic sources, and indeed disjoint activation of thalamus and neocortex has been long known^81,82^. Second, differences in total distance swum were small, in the range of tens of seconds, compared with the 5–10 min of stress used to assay stress-induced c-Fos expression.

Despite these reasons supporting a direct effect, several ambiguities remain. c-Fos expression depends in a complex and cell-type-dependent manner on activity^83^ and persists for many minutes, longer than the timescale of synaptic chains of activation (<1 s). Although a wide range of brain regions and cell types can produce c-Fos^84,85^, its synthesis is not seen in some brain regions no matter what the stimulus (for various discussions see refs. ^39,86,87^). Thus our results may be interpreted as producing a broad but not comprehensive set of candidate regions for further investigation by other methods that can unambiguously identify the effects of cerebellar perturbation with greater cell type and temporal resolution.

Lateralization of cerebellar function has previously been reported in mice for social interaction and grooming^18,88^. We observed that right crus I-targeted perturbation affected open-field behavioral habituation. Previous work found that injections targeted to either left or right crus I alone were sufficient to impair reversal learning^18^, but we found that perturbation of both sides was needed to cause statistically significant impairment. These differences could be explained if our injections had less coverage or even spillover outside targeted regions compared with previous work, which did not quantify injections. Our observation that targeted injection can cause spatially distributed expression highlights the need to quantify the spatial range of DREADD-based perturbation.

In summary, we have used chemogenetic inhibition of Purkinje cells to identify two cerebellar regions that influence multiday flexible behavior, lobule VI and crus I. Detailed characterization of c-Fos activation in Y-maze, including different stages of learning (habituation, acquisition/“nonreversal”, and reversal), revealed brainwide consequences of the task and focal perturbations. First, more challenging task conditions led to progressively more widespread regional activation. Second, cerebellar perturbation affected activity in regions that were activated under reversal learning, including thalamus by lobule VI and neocortex by crus I. Brain-wide c-Fos mapping serves as a screen analogous to the introduction of the Genome-Wide Association Study (GWAS)^89^. Understanding the task-specific role for cerebellum in driving forebrain neural processing will require going beyond “BWAS” to direct recording or high-time-resolution perturbation of the candidate regions we have identified.

## Methods

### Experimental design

We targeted neural activity of Purkinje cells of mice using inhibitory DREADDs (Fig. 1a and Supplementary Fig. 1a–c). Mice used in this study were male C57BL/6J (The Jackson Laboratory, Bar Harbor, ME) and acclimated for at least 48 h at the Princeton Neuroscience Institute vivarium prior to procedures. We stereotaxically injected virus into either lobule VI or crus I at PND 56. After a three-week recovery period, mice (PND 77) underwent behavioral testing starting with open field and then water Y-maze reversal for whole-brain c-Fos analysis (Fig. 1d and Table 1).

All mice were housed in Optimice cages (Animal Care Systems, Centennial, CO) and received environmental enrichment, including paper nesting strips and one heat-dried virgin pulp cardboard hut (Shepherd Speciality Papers, Milford, NJ). Mice were fed PicoLab Rodent Diet food pellets (LabDiet, St. Louis, MO) and water was provided ad libitum. Animals were housed in groups of 4-5 mice in reverse light cycle rooms to maximize normal nocturnal activity, as behavior testing occurred during the day. All experimental procedures were approved by the Princeton University Institutional Animal Care and Use Committee and in accordance with animal welfare guidelines of the National Institutes of Health.

### Animal preparation

We delivered adeno-associated virus (AAV) with the sequence for the inhibitory DREADD hM4Di, which was fused to mCherry protein under a Ef1α promoter. This virus included a DIO component, which when combined with the L7-cre virus, expressed in the Purkinje cell layer exclusively (Fig. 1a and Supplementary Fig. 1a–c). Recently clozapine-N-oxide (CNO) has been found to convert back to the parent compound, clozapine, in mice prior to crossing the blood-brain barrier. To reduce confounds in our experimental design, CNO was administered in control conditions where no AAV was given, as clozapine may alter signaling of neuromodulators, notably dopamine and serotonin ^90–92^.

For DREADD electrophysiology and behavioral experiments, lobules targeted were lobule VI (*n* = 36 behavior, *n* = 10 for recordings), bilateral crus I (*n* = 17), crus I right (*n* = 25), crus I left (*n* = 26), but as AAV can spread into neighboring lobules, mCherry fluorescence was recovered and quantified for the entire cerebellum. The injected fraction was calculated to the volume of the entire lobule. Controls included animals injected with AAV without DREADDs (CNO and mCherry, *n* = 18), CNO only (*n* = 30), Vehicle (DMSO and saline, *n* = 9), and untreated (*n* = 123). To understand if CNO or a lobule-specific perturbation altered Y-maze performance without reversal a subset of CNO only (*n* = 7) and CNO and Lobule VI mice (*n* = 10) underwent 25 trials of a third day of acquisition (all animals received CNO). To understand learning in the Y-maze, animals were sacrificed after habituation (n = 16), acquisition day 1 (*n* = 16), and acquisition day 2 (*n* = 10) for a total of 42 mice. When conditions allowed, the same animals went through both water Y-maze and open field testing, see Table 1. Briefly, animals were anesthetized with isofluorane (5% induction, 1–2% oxygen; 1 L/min) and mounted in a stereotaxic device (David Kopf Instrument, Tujunga, CA) for all surgeries. Temperature was monitored and automatically adjusted using PhysioSuite (Kent Scientific Corporation, Torrington, CT). Animals were prepared for surgery with an application or Puralube vet ointment (Pharmaderm Florham Park, NJ) to prevent corneal drying, the scalp was shaved and cleaned, and animals received osmotic diuretic drug 15% D-mannitol in DPBS (0.02 ml/g; intraperitoneal, i.p.) and an anti-inflammatory drug, Rimadyl (5 mg/kg Carprofen 50 mg/ml, Pfizer, Eurovet, in NaCL; every 24 h for 2 days; subcutaneous, s.c.). A lateral skin incision was made over the lambdoid suture. Muscle was cut over the occipital bones first vertically than horizontally and as close to the bone as possible to allow for regrowth post-surgery and enough to expose lobule VI or crus I. A small craniotomy was made over each lobule of interest for injection of inhibitory DREADD AAV1-Eflɑ-DIO-hM4D(Gi)-mCherry-WPRE-hGHpA (8.5 × 10^13; PNI Vector Core, AAV-VC68) or control AAV8-Eflɑ-DIO-mCherry-WPRE-hGHpA (1 × 10^15; PNI Vector Core, AAV-VC139). To target Purkinje cells, both DREADD and control AAVs were mixed in a 1:1 ratio with AAV1-sL7-Cre-HA-WPRE-hGH-pA (2 × 10^14; PNI Vector Core, AAV-VC141). Virus was injected using borosilicate glass capillaries (World Precision Instrument, Sarasota FL) made using the Sutter Micropipette Puller (Model P-2000, Sutter Instrument Company) and bevelled at a 45° angle. To ensure viral spread, ~600 nl total of DREADD or control was injected per mouse, distributed at three separate depths (150, 250, 450 µm below the dura) and two locations per lobule. Craniotomy was sealed with a silicone elastomer adhesive (Kwik-Sil, World Precision Instrument, Sarasota, Fl) and skin was sutured.

### Acute brain slice experiments

Mice (C57BL/6J) were prepared as described previously in “Animal preparation” for inhibitory DREADD induction of Purkinje cells at 3-weeks of age (n = 4). Two weeks later, mice were deeply anesthetized with Euthasol (0.06 ml/30 g), decapitated, and the brain removed. The isolated whole brains were immersed in ice-cold carbogenated NMDG ACSF solution (92 mM N-methyl D-glucamine, 2.5 mM KCl, 1.25 mM NaH_2_PO_4_, 30 mM NaHCO_3_, 20 mM HEPES, 25 mM glucose, 2 mM thiourea, 5 mM Na-ascorbate, 3 mM Na-pyruvate, 0.5 mM CaCl_2_, 10 mM MgSO_4_, and 12 mM N-acetyl-L-cysteine, pH adjusted to 7.3–7.4). Parasagittal cerebellar brain slices (270 μm) were cut using a vibratome (VT1200s, Leica Microsystems, Wetzlar, Germany), incubated in NMDG ACSF at 34 °C for 15 min, and transferred into a holding solution of HEPES ACSF (92 mM NaCl, 2.5 mM KCl, 1.25 mM NaH_2_PO_4_, 30 mM NaHCO_3_, 20 mM HEPES, 25 mM glucose, 2 mM thiourea, 5 mM Na-ascorbate, 3 mM Na-pyruvate, 2 mM CaCl_2_, 2 mM MgSO_4_, and 12 mM N-acetyl-L-cysteine, bubbled at RT with 95% O_2_ and 5% CO_2_). During recordings, slices were perfused at a flow rate of 4–5 ml/min with a recording ACSF solution (120 mM NaCl, 3.5 mM KCl, 1.25 mM NaH_2_PO_4_, 26 mM NaHCO_3_, 1.3 mM MgCl_2_, 2 mM CaCl_2_, and 11 mM D-glucose) with and without clozapine-N-oxide (CNO; 10 µM) and continuously bubbled with 95% O_2_ and 5% CO_2_. Whole-cell recordings were performed using a Multiclamp 700B (Molecular Devices, Sunnyvale, CA) using pipettes with a resistance of 3–5 MΩ filled with a potassium-based internal solution (120 mM potassium gluconate, 0.2 mM EGTA, 10 mM HEPES, 5 mM NaCl, 1 mM MgCl_2_, 2 mM Mg-ATP and 0.3 mM Na-GTP, pH adjusted to 7.2 with KOH). Purkinje neurons expressing mCherry were selected for recordings (Fig. 1e, f).

### In vivo electrophysiological recordings

Two days after performing craniotomy and headplate implantation, animals (*n* = 6) were habituated to head fixation on the treadmill. The day of the recording, cover was removed from the cranial window and then, a Neuropixels probe 1.0 (Imec, Belgium)^93^ coated with a fluorescent dye (CM-DiI, Thermofisher) was slowly inserted (0.1 mm per 1 min) using motorized micromanipulator (MP-225; Sutter Instrument Co.) into the cerebellum with the tip reaching depth of 3–5.5 mm below the brain surface. Once the right depth was reached, the probe was left to rest for 15–30 min, before starting the recording. Sterile saline was used to cover the exposed cerebellum. Signals from 384 electrodes were recorded simultaneously at 30 KHz using the Neuropixels headstage 1.0 and Neuropixels 1.0 PXIe acquisition system (Imec). High-frequencies (>300 Hz) and low-frequencies (<300 Hz) were acquired separately. SpikeGLX software (http://billkarsh.github.io/SpikeGLX/) was used to select the recording electrodes, adjust gain corrections and save data. Tactile sensory stimulation was performed in awake mice using the air puffs (40 ms, 20 psi, randomized inter-trial interval, 100 trials) delivered ipsilateral to the recording site via a small tube (2 mm diameter), approximately placed parallel to the anterior-posterior axis, 10 mm mediolateral and 1 mm anterior to the nose of the mouse, and connected to solenoid valve (The Lee Co.) controlled by paired microcontrollers (Arduino Due) and a single board computer (Raspberry Pi). Timings of air puff stimulation were digitized at 10 kHz with multifunction DAQ module (PXIe-6341 unit with BNC-2110 breakout box, National Instruments) and synchronized with using TTL pulses from PXIe acquisition module. Spikes were sorted offline using Kilosort2^94^, using default parameters. Manual curation of clusters were performed using Phy (https://github.com/cortex-lab/phy). After extracting timestamps of each putative single unit activity, peristimulus time histograms and firing rate changes were analyzed and plotted using a custom MATLAB script. DCN recording sites were identified at the time of the recording by depth and by the change or absence of units in the immediately overlying white matter and later confirmed by post-hoc histology in 100 µm coronal cerebellar sections recording tracks were identified with CM-DiI marks (C7001, ThermoFisher Scientific, MA, USA) (Fig. 1g and Supplementary Fig. 2).

### Tissue processing and histology

To examine mCherry fluorescence, the presence of DREADD virus, two mice were anesthetized with Euthasol (0.06 ml/30 g, i.p.) and perfused with 4% paraformaldehyde (PFA). Brains were stored overnight at 4% PFA then placed in 20% sucrose in PBS overnight until sectioning at 50 µm. Sections were washed with PBS and incubated for 1 h at room temperature (RT) in blocking buffer (10% normal donkey serum, 0.5% Triton in PBS) prior to an overnight incubation at 4°C in PBS buffer with 2% normal donkey serum, 0.4% Triton and the rabbit anti-RFP (600-401-379, Rockland Immunochemicals, Inc., Limerick, PA; 1:1000) primary antibody. The next day, sections were washed in PBS and incubated for 2 hr at RT in PBS buffer with 2% normal donkey serum, 0.4% Triton, and the donkey anti-Rabbit Alexa Fluor 647-conjugated secondary antibody (A-21449; Thermo Fisher Scientific, MA, USA, Invitrogen; 1:400).Tissue was mounted on glass slides with Prolong Diamond (ThermoFisher Scientific, MA, USA). Tissue was imaged with a Leica SP8 confocal laser-scanning microscope (Leica Microsystems, Germany) using ×40 or ×63 objectives (Supplementary Fig. 1b-c). To confirm tissue clearing and lightsheet microscopy, brains were sectioned and stained using conventional immunohistochemistry. Free-floating tissue sections were cut using a vibratome (VT-1000S, Leica Microsystems, Germany). After washing in phosphate-buffered saline (PBS) ph 7.6 at RT for four 10 min sessions, sections were blocked with 10% donkey serum and 0.5% Triton X-100 in PBS for 1 h. Post-incubation, sections were placed in the primary solution of rabbit anti-Fos (226 003, Synaptic Systems,Goettingen, Germany, 1:1000) with 2% donkey serum and 0.5% Triton X-100 in PBC for 48 h at 4 °C. Sections were washed for three 10 min sessions in PBS then placed in secondary donkey anti-Rabbit Alexa Fluor 647-conjugated secondary antibody (A-21449; Thermo Fisher Scientific, MA, USA, Invitrogen; 1:200) in 2% donkey serum and 0.4% Triton X-100 in PBS for 2 h at RT. Sections were washed for three 10 min sessions in PBS. Tissue was imaged at ×20 using an epifluorescence microscope (NanoZoomer S60, Hamamatsu Photonics) and c-Fos positive cells were counted in ImageJ using the “Analyze Particles” function (Supplementary Fig. 8a and d). After animals completed the last Y-maze reversal session, animals were placed back in their home cage for 90 min. Then mice were anesthetized with Euthasol (0.06 ml/30 g, i.p.) and perfused with 4% PFA for analysis of c-Fos and mCherry expression for DREADD recovery using iDISCO+ clearing methods^5,41,42^. Briefly, after an overnight fix in 4% PFA, brains were rinsed in PBS at RT for four 30 min sessions. Immediately brains were dehydrated 1 h at each ascending concentration of methanol (20, 40, 60, 80, 100, 100%) and placed overnight in 5% hydrogen peroxide and methanol at RT. The next day, brains were rehydrated for 1 hr at each descending concentration of methanol (100, 80, 60, 40, 20%) and lastly PBS. Samples were placed in detergent (0.2% Triton X-100 in PBS) for two 1 h sessions then placed for 2 days in 20% Dimethyl sulfoxide (DMSO), 0.3 M glycine, 0.2% Triton X-100 in PBS at 37 °C. Brains were blocked in 10% DMSO, 6% donkey serum, 0.2% Triton X-100 in PBS at 37 °C for 3 days. Once at RT, samples were washed in PTwh (0.2% Tween-20, 10 µg/ml heparin in PBS) and placed in primary solution of rabbit anti-Fos (226 003, Synaptic Systems, Goettingen, Germany, 1:1000) and/or rabbit anti-RFP (600-401-379, Rockland Immunochemicals, Inc., Limerick, PA; 1:450) for one week at 37°C. Brains were washed in PTwH five times in increasing amounts of time (10, 15, 30, 60, 120 min) and then placed in secondary donkey anti-Rabbit Alexa Fluor 647-conjugated secondary antibody (A-21449; Thermo Fisher Scientific, MA, USA, Invitrogen; 1:200) for 1 week at 37 °C. Brains were washed in PTwH five times in increasing amounts of time (10, 15, 30, 60, 120 min) and then dehydrated 1 h at each ascending concentration of methanol (20, 40, 60, 80, 100, 100%) until being placed in 66% dichloromethane (DCM)/33% methanol for 3 h at RT. Brains were cleared with 100% DCM for two 15 min steps and then placed in 100% benzyl ether (DBE). Brains were kept in fresh DBE prior to imaging on a lightsheet microscope and after for long-term storage.

### Water Y-maze

The water Y-maze assay and analysis were similar to described^16^. Briefly, the Y-shaped transparent polycarbonate apparatus had symmetrical arms, each measuring 33 cm × 7.5 cm × 20.3 cm (length × width × height) from the center. Notches in all three arms (9.5 cm from the center) allowed for a removable gate. A Pyrex glass container was used as a platform for the mice to climb on. To prevent the animals from seeing the platform, opaque water was used by mixing ACMI-certified hypoallergenic non-toxic white paint (Art-Minds, Tempera Paint) in warm water. Water levels were kept at a depth to prevent the animals from touching the bottom of the maze. Between each mouse, excrement was removed and water was exchanged to maintain an ideal water temperature between 22 and 28°C. At the end of the day, water was removed for cleaning by PREempt disinfectant wipes (0.5% Hydrogen Peroxide) and sprayed down with 70% ethanol to be left overnight to dry. To prevent distraction, a three-walled shield was placed around the maze constructed of non-reflective black plastic (34 × 29 × 22 cm, length x width x height). Directly above the arena, a camera (PlayStation Eye) was mounted on a T-slotted aluminum rail (80/20 KNOTTS Company, Berkeley Heights, NJ) and used to record the entire field of view at 50 frames/s using a custom Python 2.7.6 script (Anaconda 1.8.0) and a CLEye Driver (https://codelaboratories.com/products/eye/driver/).

Over four consecutive days animals were habituated to the arena (Day 1; three 60 s trials), learned to find a platform through trial and error (Acquisition Day 2 and Day 3; four sessions with five 40 s trials), then exposed to reversal whereby the platform was moved to the opposite arm the animals learned (Day 4; five sessions with five 40 s trials) (Fig. 2a). Mice were required to have 80% success rate for acquisition day 3 in order to progress to the reversal day. During the reversal day, animals were exposed to four sessions of five consecutive trials followed by a fifth forced session whereby a door was placed in the initial learned arm of the maze which no longer has a platform. All mice were kept in a clean cage on a heating pad to dry before returning to their homecage. As the temperature difference between the warming cage and the water can be drastic, it is critical to allow the animal to cool down prior to starting a new session. Performance on first-choice turn direction in the Y-maze assay was calculated automatically. Neither surgery by itself, nor the effects of administering vehicle-only, CNO-without-DREADD, or CNO-with-mCherry, affected distance swum, initial learning, or reversal-learning compared with untreated mice (Supplementary Fig. 3a–c). Distance traveled during habituation was calculated to assess possible muscle damage during surgery (Supplementary Fig. 3a). Subsequent tries were recorded to calculate the fraction of choices required to reach the platform (Fig. 2d and Supplementary Fig. 3f).

### Lightsheet microscopy

Briefly, cleared brains were glued (Loctite, 234796) ventral-side-down to a 3D-printed holder and imaged in DBE. Brains were registered using the autofluorescence channel (488 nm laser diode excitation and 525 emission) to the Princeton Mouse Atlas. Cellular imaging of c-Fos and mCherry expression was acquired using 640 nm excitation and 680 nm emission (×1 magnification, ×1.3 objective, 0.1 numerical aperture, 9.0 mm working distance, 12.0 × 12.0 mm field of view, LVMI-Fluor 1.3x, LaVision Biotech) with a 10 μm step-size using a 0.016 excitation NA. Analysis of whole-brain c-Fos was completed using ClearMap^43^ on high-performance computing clusters. To confirm ClearMap cell counts, two human annotators analyzed 14 brain volumes and found 96.72% correspondence between cells counted by the human annotators and ClearMap-counted cells (Supplementary Fig. 4c). Structures less than 80 microns and structures in the medulla were removed from analysis. The medulla was not analyzed as it can be damaged during brain extraction. Ventricles, brain edges, and zones within 60 microns of region boundaries were removed. The cerebellum was not analyzed for c-Fos activity. Visualization of brain volumes and cell detections from ClearMap was performed using Neuroglancer (Supplementary Fig. 4a, b). Tissue image processing and registration were performed using custom Python code (https://github.com/PrincetonUniversity/BrainPipe).

We assessed the impact of varying experimental conditions in the following contrasts:

1. Acquisition day 1 vs Habituation
2. CNO control reversal vs CNO control no reversal
3. CNO control reversal vs Vehicle control reversal
4. Vector control reversal vs Vehicle control reversal
5. Lobule VI reversal vs CNO control reversal
6. Crus I left vs CNO control reversal
7. Crus I right vs CNO control reversal
8. Bilateral Crus I vs CNO control reversal
9. Lobule VI reversal vs Lobule VI no reversal

In contrasts 1-5, both the control and the treatment groups of mice were processed in the same batch so as to minimize batch effects. In contrasts 6-7, both control and treatment groups were present in multiple batches, so batch (encoded as a categorical indicator variable) was included as a covariate to adjust for the batch effect. Finally, in contrasts 8-9 control and treatment groups did not overlap within any single batch, impeding the direct adjustment for confounding due to batch effects. However, we were able to adjust for confounding indirectly by using a “bridge variable” strategy inspired by the chain-type experimental design described by Song et al^95^. We define a bridge variable as an experimental group found in both the batch containing treatment animals as well as the batch containing control animals. The bridge variables were Lobule VI reversal for contrast 8 and CNO control reversal for contrast 9. Inclusion of the bridge variable along with the batch indicator variables in a regression model makes the treatment versus control contrast statistically identifiable separate from the batch effect, as previously shown^95^.

Total counts of active neurons were highly variable between animals even in the same experimental condition. We therefore sought to explain differences in proportion of total counts for each brain region. Given the overdispersed, discrete nature of the data, we used a negative binomial likelihood and performed separate regressions for each brain region and contrast, using the natural log of total counts as an offset. Specifically, for a given contrast, let *X*_i_ = 1 if animal i is in the treatment group and *X*_i_ = 0 otherwise. Let *Z*_i_ = 1 if the animal is in the first batch and *Z*_i_ = 0 otherwise. Let *A*_i_ = 1 if the animal has the bridge variable condition and *A*_i_ = 0 otherwise. Let T_i_ be the total c-Fos counts across all regions in brain i. Let *Y*_ij_ be the c-Fos count in region j of brain i. The statistical model is then given by

*Y*_ij_ ~ negative binomial (*μ*_ij_,*φ*_j_)

$${{\log }}\left({{{{{\rm{\mu }}}}}}{{{{{\rm{ij}}}}}}\right)={{{{{{\rm{\beta }}}}}}}_{0{{{{{\rm{j}}}}}}}+{{{{{{\rm{X}}}}}}}_{{{{{{\rm{i}}}}}}}^{* }{{{{{{\rm{\beta }}}}}}}_{1{{{{{\rm{j}}}}}}}+{{{{{\rm{ln}}}}}}\left({{{{{{\rm{T}}}}}}}_{{{{{{\rm{i}}}}}}}\right)+{{{{{{\rm{Z}}}}}}}_{{{{{{\rm{i}}}}}}}^{* }{{{{{{\rm{\beta }}}}}}}_{2{{{{{\rm{j}}}}}}}+{{{{{{\rm{A}}}}}}}_{{{{{{\rm{i}}}}}}}^{* }{{{{{{\rm{\beta }}}}}}}_{3{{{{{\rm{j}}}}}}}$$

where *μ*_ij_ is the expected value of *Y*_ij_, *φ*_j_ is the nuisance shape parameter of the negative binomial distribution, and *β*_0j_ is an intercept term that captures the fact that some brain regions have a consistently higher or lower activity level across all animals without regard to their experimental condition (for example this could be because some regions have a larger volume than others). The parameter *β*_1j_ is of greatest biological interest. It is interpreted as the average change in proportion of c-Fos activity for region j in the treatment group relative to the control group on the logarithmic scale. For example, if *β*_1j_ = 1.5, the average c-Fos activity for brain region j is estimated to be exp(1.5) = 4.48 times higher in the treatment condition compared to the control condition. If *β*_1j_ = −0.7 (a negative coefficient) average c-Fos activity for brain region j is estimated to be exp(−0.7) = 0.5 times lower in the treatment condition compared to the control (ie, the treatment mean is half that of the control mean). We fit each negative binomial regression using the R package MASS^96^. In addition to maximum likelihood estimates of the regression coefficients such as *β*_1j_, this package also computes a standard error for each coefficient. From these, we obtained effect sizes in the form of Wald z-test statistics (estimated coefficient divided by standard error). Under the null hypothesis that there is no change in the average c-Fos expression between the treatment and control groups (i.e. *β*_1j_ = 0), the effect sizes would be expected to follow an asymptotically standard normal distribution. By comparing the computed effect sizes against this null distribution, we obtained p-values. If the *p* value was small for a given brain region, it suggested that there was a large difference between the treatment and control groups and the null hypothesis should be rejected for that region. Since a separate statistical test was performed for each brain region, we adjusted raw p-values in each analysis to control the multiple testing false discovery rate (FDR) using the method of Benjamini and Hochberg^97^.

In rare cases, numerical errors occurred in the MASS package fitting procedure. This is because the estimation of the negative binomial parameters (*β*_0j_, *β*_1j_, *β*_2j_, *β*_3j_, and *φ*_j_) requires an iterative optimization that can fail to converge. We determined that in most of these cases, the brain region was small and/or irregularly shaped. We, therefore, dropped such regions from subsequent analysis.

In contrast 8 only, some observations came from batch 201810_adultacutePC_ymaze_cfos, in which brains had cerebellum excised. As a quality control step, we excluded regions with extremely low counts and low variability across all brains in this batch since this would destabilize the regression fitting procedure. Specifically, for each region we counted the number of brains within the batch having a nonzero count value. If the number of nonzero counts was less than two, we excluded the region.

In addition to analyzing individual brain regions, we also fit negative binomial regressions to examine the effects of experimental perturbations on three composite regions consisting of multiple subregions from our original 122 regions: thalamus, sensory/motor, and polymodal association (Supplementary Data 1). For each composite region and experimental contrast, we summed the raw c-Fos cell counts of all constituent subregions within each animal. We then fit negative binomial regressions as previously described, with the natural log of the total counts in the entire brain again used as an offset for each animal. This maintained consistency in the interpretation of the regression coefficients and effect sizes by keeping them on the same (proportional) scale as the original analysis. To confirm our c-Fos statistical results, we ran two sample t-tests for each region per condition comparison, and then checked the validity of the resulting p-values using permutation testing. None of the highly significant regions from the negative binomial regression were missing.

For functional network analysis, pairs of brain regions were tested for correlation by calculating the cell counts (normalized to whole-brain count) on a mouse-by-mouse basis, and using those to calculate a Spearman correlation ρ across all M mice. A pair of brain regions was selected for plotting a connected line if *ρ* ≥ *ρ*_threshold_, which was set at 0.4, or higher as needed to reach a false discovery rate of 0.05, using the following procedure: P-values were calculated using a Fisher z-transformation as a two-tailed *P* value *P* = 2*normcdf(-atanh(ρ_threshold_)*√(*M*−3)), where normcdf(*x*) is the area under the normal curve less than *x* and atanh() is the inverse hyperbolic tangent^98^. The resulting distribution of *P* values was used to estimate the proportion of null values, *π*_0_, using a tuning parameter of *λ* = 0.5^99^. Taking *C* as the number of connections detected in all comparisons between pairs of k regions with *P* < *P*_threshold_, the false discovery rate was calculated as FDR= *P*_threshold_*π*_0_(*k*−1)(*k*−2)/2*C* (Supplementary Data 2). For setting *ρ*_threshold_, regions showing up as different in the BWAS analysis were used. The exception to this was the vehicle control group (part of the vehicle vs. CNO comparison), for which all regions were used.

### Open field

Wild-type (*n* = 60)^50^, mice with a cerebellar perturbation (*n* = 10 per group) and controls, including CNO only (*n* = 12), CNO and mCherry (*n* = 10), and vehicle only (*n* = 10). Previous data collected^50^ using Purkinje-cell specific tuberous sclerosis 1 gene mutation (L7Cre; Tsc1^flox/flox^) (*n* = 9) was used as a comparison for gait analysis (Supplementary Fig. 19b). Animals were placed in an open field arena measuring 45.72 × 45.72 cm (length × width) and 30.48 cm in height with a transparent polycarbonate floor, as previously described^50^. A Point Grey grayscale camera (12-bit grayscale, 1280 × 1024 pixel resolution at 80 Hz) was used to image from below. The soundproof box with ventilation was illuminated with far-red LEDs. To prevent noise disturbance, doors were kept closed during acquisition. Mice received CNO on day 1, if applicable, and vehicle on day 2 to understand how perturbation may alter open field habituation. Each mouse was recorded for 20 min before returning to group housing over two days (first 18 min 46 s are included in the analysis). Raw images were processed to segment the mouse from the background (median filter) to a final video size of 400 × 400 pixels.

### Machine learning

Each frame was aligned for the mouse body axis and body parts were tracked using LEAP (LEAP Estimates Animal Poses) as previously described^50,51^. The LEAP network was trained on 1000 frames to find 18 body parts. Automatic classification of animal behavior was performed using custom MATLAB and Python scripts as previously described^50^. Briefly, distances between 11 body parts (nose, chin, 4 x paw tip, 4 x paw base, where paw connects to leg, and tail base) were calculated and the dimensionality was reduced by projecting on the first 10 PCA components. A wavelet analysis was performed in the lower dimensional space, followed by *k*-means clustering (*k* = 100) of the frequency data to obtain behavioral clusters. The 100 behavioral clusters were manually grouped into 6 behaviors (slow explore, grooming, fast explore, reared, turning walk, locomotion) (Fig. 7b). A majority filter with a sliding window of 11 frames was used on the predicted behaviors for each frame. Centroid metrics were used to calculate distance traveled, and fraction of time in the inner arena. The inner part of the arena was defined as the region of the arena with a distance larger than 150 pixels (~7.6 cm) to all borders of the arena. The borders were obtained using the space explored by the mouse. To calculate the temporal change of the probability to be in fast locomotion within the time of an experiment, a sliding window of size 20001 frames was used. The probabilities at equally distant time points were calculated for each mouse separately, based on which the mean and standard deviation were obtained (Fig. 7c).

For the shifts in state occupancy, the fractions of time spent in each behavior during the first 5 min and the remainder of the experiment were calculated for each mouse. Then, the geometric mean was taken for each group and day and the geometric mean values for day 2 were divided by the ones from day 1 for each group (Fig. 7d). The shifts in state transitions were obtained as follows: First, the transition matrices were calculated for each mouse and day by considering only changes of states as transitions (i.e. the transition rate to stay in the same state is zero). Then, the transition matrices are averaged for each group and day by weighting the transition probabilities from initial state i by the probability of the mouse to be in state i. Finally, the resulting averaged transition matrix for day 1 is subtracted from the one for day 2 for each group, which allows an investigation of the changes in state transitions across days. To study the possibility of gait changes in cerebellar perturbed mice compared to controls, the phases when the paws enter stance during a single stride of locomotion were calculated for different centroid velocities^57^. The beginning of the stance phase was determined by the peak position of the paw in an animal-centered coordinate system. All analyses of the open field behavioral data were performed using custom Matlab code: (https://github.com/PrincetonUniversity/OF-ymaze-cfos-analysis).

### Statistics and reproducibility

All statistics were performed using MATLAB, R (rstatix, compositions, npmv, data.table, plyr, ggplot2, ggpubr, car, DescTools), or Python 2 and Python 3 (statsmodels, scipy, matplotlib, numpy). Data are presented as mean ± SD, unless otherwise stated. Group mean comparisons were performed through repeated measures ANOVA (open field) or through one-way ANOVAs with Tukey HSD or Dunnett multiple comparisons post-hoc tests. For each comparison, the effect size (Cohen’s *d*) or Kolmogorov-Smirnov test was calculated. Normality was tested using the Shapiro-Wilk test, and the homogeneity of variances was tested using Levene’s test to determine parametric or nonparametric analysis. If the Shapiro–Wilk test or Levene’s test was significant, the nonparametric Kruskal-Wallis test was performed, followed by pairwise comparisons with Wilcoxon rank sum exact tests (with Benjamini–Hochberg multiple comparison correction). In this case, the Wilcoxon effect size r was calculated for each comparison. Y-maze performance was measured by the number of successes and failures of each mouse in five trials for different sessions and days. To account for the fact that the data were nested and can take only values between 0 and 100% (more specifically 0, 20, 40, 60, 80, and 100%), we fit GLMM with a binomial distribution and logit link function to the performance data for acquisition day 2, 3, and reversal day (using glmer function in R package lme4^100^. We included the session and the experimental group as fixed effects predictors. Since the performance scores within mice may be correlated, we also incorporated random intercepts in the model. We tested different models (with and without the interaction term of group and session; considering session as a factor or a quantitative variable; with and without random slopes added to random intercepts) and chose the models based on the Akaike information criterion and likelihood ratio tests. The data for acquisition day 2 and day 3 was best described by a model without the interaction term of group and session and random intercepts only. To test for significant differences in the performance of the experimental groups, we performed multiple comparisons of the means (Dunnett contrasts, using the glht function in the R package multcomp)^101^. For the reversal day, we also considered a model that accounts for interactions of group and session and tested for within-session differences between the groups (Bonferroni-Holm multiple comparison correction).

The number of structures used for c-Fos analysis were tested for multiple comparisons by calculating the false discovery rate, the coefficient of variation, and by performing permutation tests on each comparison. All c-Fos data are presented as *p* < 0.01 unless otherwise stated. To analyze correlations between whole-brain c-Fos/DREADD and behavior, Spearman’s rank correlation coefficient (ρ) and p-values were determined using stats models in Python 3 or Matlab. Correlations between brain regions were visualized using network modeling in Matlab.

To investigate differences between the behaviors of mice in the open field we performed a compositional data analysis to account for the compositional nature of the data^102^. First, for each mouse, the set of fractions of time spent in each behavior was transformed into isometric log ratio coordinates using the R package “compositions”. In this coordinate space, differences between groups were analyzed using a nonparametric multivariate test (Wilks’ Lambda type test statistics) from the R package “npmv”. To identify the behaviors that differ between groups, we calculated the bootstrapped 95% confidence intervals (*N* = 5000) of the log ratio differences between the cerebellar perturbed groups and mice without a cerebellar perturbation given CNO on day 1 for each behavior^102^. The same compositional data analysis was performed on the different control groups using the geometric mean values of all control groups combined as a reference group. The Matlab and R code used for the statistical analyses is published on Github: (https://github.com/PrincetonUniversity/OF-ymaze-cfos-analysis).

### Reporting summary

Further information on research design is available in the Nature Portfolio Reporting Summary linked to this article.

## Supplementary information

Supplementary Information

Description of Additional Supplementary Files

Supplementary Data 1

Supplementary Data 2

Supplementary Movie 1

Supplementary Movie 2

Supplementary Movie 3

Supplementary Movie 4

Reporting Summary

## Data Availability

The dataset is available at Princeton data 10.34770/c9df-sc15 and https://brainmaps.princeton.edu/2022/01/verpeut-et-al-data-exploration-links/.

## Code availability

The code is freely available at https://github.com/PrincetonUniversity/OF-ymaze-cfos-analysis and 10.34770/c9df-sc15.

## References

[1] Leiner HC, Leiner AL, Dow RS (1986). Does the cerebellum contribute to mental skills?. Behav. Neurosci..

[2] Leiner HC, Leiner AL, Dow RS (1993). Cognitive and language functions of the human cerebellum. Trends Neurosci..

[3] De Zeeuw CI, Lisberger SG, Raymond JL (2021). Publisher Correction: Diversity and dynamism in the cerebellum. Nat. Neurosci..

[4] De Zeeuw CI (2021). Bidirectional learning in upbound and downbound microzones of the cerebellum. Nat. Rev. Neurosci..

[5] Pisano TJ (2021). Homologous organization of cerebellar pathways to sensory, motor, and associative forebrain. Cell Rep..

[6] Schmahmann JD, Sherman JC (1998). The cerebellar cognitive affective syndrome. Brain.

[7] Schmahmann, J. D. Cerebellar cognitive affective syndrome and the neuropsychiatry of the cerebellum. *Handbook of the Cerebellum and Cerebellar Disorders*. pp. 1717–1751 (Cham, Springer International Publishing, 2021).

[8] Limperopoulos C, Chilingaryan G, Guizard N, Robertson RL, Du Plessis AJ (2010). Cerebellar injury in the premature infant is associated with impaired growth of specific cerebral regions. Pediatr. Res..

[9] Wang SS-H, Kloth AD, Badura A (2014). The cerebellum, sensitive periods, and autism. Neuron.

[10] Limperopoulos C (2014). Injury to the premature cerebellum: outcome is related to remote cortical development. Cereb. Cortex.

[11] Uddin LQ (2015). Brain state differentiation and behavioral inflexibility in autism. Cereb. Cortex.

[12] Latinus M (2019). Inflexibility in autism spectrum disorder: need for certainty and atypical emotion processing share the blame. Brain Cogn..

[13] Fatemi SH (2012). Consensus paper: pathological role of the cerebellum in autism. Cerebellum.

[14] Crucitti J, Hyde C, Enticott PG, Stokes MA (2020). Are vermal lobules VI-VII smaller in autism spectrum disorder?. Cerebellum.

[15] Traut N (2018). Cerebellar volume in autism: literature meta-analysis and analysis of the autism brain imaging data exchange cohort. Biol. Psychiatry.

[16] Badura A (2018). Normal cognitive and social development require posterior cerebellar activity. Elife.

[17] Sugihara I (2018). Crus I in the rodent cerebellum: its homology to Crus I and II in the primate cerebellum and its anatomical uniqueness among neighboring lobules. Cerebellum.

[18] Stoodley CJ (2017). Altered cerebellar connectivity in autism and cerebellar-mediated rescue of autism-related behaviors in mice. Nat. Neurosci..

[19] Deverett B, Kislin M, Tank DW, Wang SS-H (2019). Cerebellar disruption impairs working memory during evidence accumulation. Nat. Commun..

[20] Deverett, B., Koay, S. A., Oostland, M. & Wang, S. S.-H. Cerebellar involvement in an evidence-accumulation decision-making task. *eLife* **7**, e36781 (2018). 10.7554/eLife.36781 PMC6105309 30102151

[21] Strick PL, Dum RP, Fiez JA (2009). Cerebellum and nonmotor function. Annu. Rev. Neurosci..

[22] Kelly RM, Strick PL (2003). Cerebellar loops with motor cortex and prefrontal cortex of a nonhuman primate. J. Neurosci..

[23] Eccles JC, Llinás R, Sasaki K (1966). The excitatory synaptic action of climbing fibres on the Purkinje cells of the cerebellum. J. Physiol..

[24] Hull, C. & Regehr, W. G. The cerebellar cortex. *Annu. Rev. Neurosci.* **45**, 151–175 (2022). 10.1146/annurev-neuro-091421-125115 PMC10268027 35803588

[25] Andersson G, Oscarsson O (1978). Climbing fiber microzones in cerebellar vermis and their projection to different groups of cells in the lateral vestibular nucleus. Exp. Brain Res..

[26] Apps R, Hawkes R (2009). Cerebellar cortical organization: a one-map hypothesis. Nat. Rev. Neurosci..

[27] Gravel C, Hawkes R (1990). Parasagittal organization of the rat cerebellar cortex: direct comparison of Purkinje cell compartments and the organization of the spinocerebellar projection. J. Comp. Neurol..

[28] Gao Z (2018). A cortico-cerebellar loop for motor planning. Nature.

[29] Chabrol FP, Blot A, Mrsic-Flogel TD (2019). Cerebellar contribution to preparatory activity in motor neocortex. Neuron.

[30] Khalil AJ, Mansvelder HD, Witter L (2022). Mesodiencephalic junction GABAergic inputs are processed separately from motor cortical inputs in the basilar pons. iScience.

[31] McAfee SS, Liu Y, Sillitoe RV, Heck DH (2021). Cerebellar coordination of neuronal communication in cerebral cortex. Front. Syst. Neurosci..

[32] Wagner MJ (2019). Shared cortex-cerebellum dynamics in the execution and learning of a motor task. Cell.

[33] Wang X, Novello M, Gao Z, Ruigrok TJH, De Zeeuw CI (2022). Input and output organization of the mesodiencephalic junction for cerebro-cerebellar communication. J. Neurosci. Res..

[34] Sillitoe RV, Künzle H, Hawkes R (2003). Zebrin II compartmentation of the cerebellum in a basal insectivore, the Madagascan hedgehog tenrec Echinops telfairi. J. Anat..

[35] Reeber SL, White JJ, George-Jones NA, Sillitoe RV (2012). Architecture and development of olivocerebellar circuit topography. Front. Neural Circuits.

[36] Ozol K, Hayden JM, Oberdick J, Hawkes R (1999). Transverse zones in the vermis of the mouse cerebellum. J. Comp. Neurol..

[37] Oostland, M. et al. Enhanced learning and sensory salience in a cerebellar mouse autism model. *bioRxiv* 2021.12.23.474034 10.1101/2021.12.23.474034 (2021).

[38] Tsai PT (2018). Sensitive periods for cerebellar-mediated autistic-like behaviors. Cell Rep..

[39] Dragunow M, Faull R (1989). The use of c-fos as a metabolic marker in neuronal pathway tracing. J. Neurosci. Methods.

[40] Kovács KJ (1998). c-Fos as a transcription factor: a stressful (re)view from a functional map. Neurochem. Int..

[41] Renier N (2014). iDISCO: a simple, rapid method to immunolabel large tissue samples for volume imaging. Cell.

[42] Pisano TJ (2022). Automated high-throughput mouse transsynaptic viral tracing using iDISCO+ tissue clearing, light-sheet microscopy, and BrainPipe. STAR Protoc..

[43] Renier N (2016). Mapping of brain activity by automated volume analysis of immediate early genes. Cell.

[44] Tian JB, Bishop GA (2002). Stimulus-dependent activation of c-Fos in neurons and glia in the rat cerebellum. J. Chem. Neuroanat..

[45] Jones, E. G. *The Thalamus*. Springer Science & Business Media (2012).

[46] Burton H, Jones EG (1976). The posterior thalamic region and its cortical projection in New World and Old World monkeys. J. Comp. Neurol..

[47] Tanimizu T (2017). Functional connectivity of multiple brain regions required for the consolidation of social recognition memory. J. Neurosci..

[48] Durieux L (2022). Functional brain-wide network mapping during acute stress exposure in rats: Interaction between the lateral habenula and cortical, amygdalar, hypothalamic and monoaminergic regions. Eur. J. Neurosci..

[49] Storey, J. D. & Tibshirani, R. SAM Thresholding and false discovery rates for detecting differential gene expression in DNA microarrays. In: *The Analysis of Gene Expression Data: Methods and Software* (eds. Parmigiani, G., Garrett, E. S., Irizarry, R. A. & Zeger, S. L.) 272–290 (Springer, New York, 2003). 10.1007/0-387-21679-0_12.

[50] Klibaite U (2022). Deep phenotyping reveals movement phenotypes in mouse neurodevelopmental models. Mol. Autism.

[51] Pereira TD (2019). Fast animal pose estimation using deep neural networks. Nat. Methods.

[52] Pereira TD (2022). SLEAP: a deep learning system for multi-animal pose tracking. Nat. Methods.

[53] Wiltschko AB (2015). Mapping sub-second structure in mouse behavior. Neuron.

[54] Wiltschko AB (2020). Revealing the structure of pharmacobehavioral space through motion sequencing. Nat. Neurosci..

[55] Mathis A (2018). DeepLabCut: markerless pose estimation of user-defined body parts with deep learning. Nat. Neurosci..

[56] Nath T (2019). Using DeepLabCut for 3D markerless pose estimation across species and behaviors. Nat. Protoc..

[57] Machado AS, Darmohray DM, Fayad J, Marques HG, Carey MR (2015). A quantitative framework for whole-body coordination reveals specific deficits in freely walking ataxic mice. Elife.

[58] Tsai PT (2012). Autistic-like behaviour and cerebellar dysfunction in Purkinje cell Tsc1 mutant mice. Nature.

[59] Maes JHR, Eling PATM, Wezenberg E, Vissers CTWM, Kan CC (2011). Attentional set-shifting in autism spectrum disorder: differentiating between the role of perseveration, learned irrelevance, and novelty processing. J. Clin. Exp. Neuropsychol..

[60] Sasson NJ, Turner-Brown LM, Holtzclaw TN, Lam KSL, Bodfish JW (2008). Children with autism demonstrate circumscribed attention during passive viewing of complex social and nonsocial picture arrays. Autism Res.

[61] Reese RM, Richman DM, Zarcone J, Zarcone T (2003). Individualizing functional assessments for children with autism: the contribution of perseverative behavior and sensory disturbances to disruptive behavior. Focus Autism Other Dev. Disabl..

[62] Caulfield MD, Zhu DC, McAuley JD, Servatius RJ (2016). Individual differences in resting-state functional connectivity with the executive network: support for a cerebellar role in anxiety vulnerability. Brain Struct. Funct..

[63] Ottaviani C (2016). Neurobiological substrates of cognitive rigidity and autonomic inflexibility in generalized anxiety disorder. Biol. Psychol..

[64] Schmahmann JD (2004). Disorders of the cerebellum: ataxia, dysmetria of thought, and the cerebellar cognitive affective syndrome. J. Neuropsychiatry Clin. Neurosci..

[65] Hu H, Cui Y, Yang Y (2020). Circuits and functions of the lateral habenula in health and in disease. Nat. Rev. Neurosci..

[66] Andres KH, von Düring M, Veh RW (1999). Subnuclear organization of the rat habenular complexes. J. Comp. Neurol..

[67] Chiang MC (2019). Parabrachial complex: a Hub for pain and aversion. J. Neurosci..

[68] Deng H, Xiao X, Wang Z (2016). Periaqueductal gray neuronal activities underlie different aspects of defensive behaviors. J. Neurosci..

[69] Capuzzo G, Floresco SB (2020). Prelimbic and infralimbic prefrontal regulation of active and inhibitory avoidance and reward-seeking. J. Neurosci..

[70] Boulougouris V, Dalley JW, Robbins TW (2007). Effects of orbitofrontal, infralimbic and prelimbic cortical lesions on serial spatial reversal learning in the rat. Behav. Brain Res..

[71] Ghahremani DG, Monterosso J, Jentsch JD, Bilder RM, Poldrack RA (2010). Neural components underlying behavioral flexibility in human reversal learning. Cereb. Cortex.

[72] Chao, O. Y. et al. Social memory deficit gated by dysregulation of the cerebellar vermis. 10.21203/rs.3.rs-1393639/v1 (2022).

[73] Fujita H, Kodama T, du Lac S (2020). Modular output circuits of the fastigial nucleus for diverse motor and nonmotor functions of the cerebellar vermis. Elife.

[74] Halassa MM, Kastner S (2017). Thalamic functions in distributed cognitive control. Nat. Neurosci..

[75] Houser CR, Vaughn JE, Barber RP, Roberts E (1980). GABA neurons are the major cell type of the nucleus reticularis thalami. Brain Res.

[76] Harting JK, Van Lieshout DP, Feig S (1991). Connectional studies of the primate lateral geniculate nucleus: distribution of axons arising from the thalamic reticular nucleus of Galago crassicaudatus. J. Comp. Neurol..

[77] Guillery RW, Harting JK (2003). Structure and connections of the thalamic reticular nucleus: advancing views over half a century. J. Comp. Neurol..

[78] Cavdar S (2002). Cerebellar connections to the rostral reticular nucleus of the thalamus in the rat. J. Anat..

[79] De Zeeuw CI, Lisberger SG, Raymond JL (2021). Diversity and dynamism in the cerebellum. Nat. Neurosci..

[80] Saper, C. B. Hypothalamic connections with the cerebral cortex. *Prog. Brain Res.* **126**, 39–48 (2000). 10.1016/S0079-6123(00)26005-6 11105638

[81] Vanderwolf CH, Stewart DJ (1988). Thalamic control of neocortical activation: a critical re-evaluation. Brain Res. Bull..

[82] Fuller PM, Sherman D, Pedersen NP, Saper CB, Lu J (2011). Reassessment of the structural basis of the ascending arousal system. J. Comp. Neurol..

[83] Fields RD, Eshete F, Stevens B, Itoh K (1997). Action potential-dependent regulation of gene expression: temporal specificity in ca2+, cAMP-responsive element binding proteins, and mitogen-activated protein kinase signaling. J. Neurosci..

[84] Sheng M, Greenberg ME (1990). The regulation and function of c-fos and other immediate early genes in the nervous system. Neuron.

[85] Cullinan WE, Herman JP, Battaglia DF, Akil H, Watson SJ (1995). Pattern and time course of immediate early gene expression in rat brain following acute stress. Neuroscience.

[86] Chan RK, Sawchenko PE (1994). Spatially and temporally differentiated patterns of c-fos expression in brainstem catecholaminergic cell groups induced by cardiovascular challenges in the rat. J. Comp. Neurol..

[87] Holstein GR (2012). Fos expression in neurons of the rat vestibulo-autonomic pathway activated by sinusoidal galvanic vestibular stimulation. Front. Neurol..

[88] Kelly E (2020). Regulation of autism-relevant behaviors by cerebellar-prefrontal cortical circuits. Nat. Neurosci..

[89] Ozaki K (2002). Functional SNPs in the lymphotoxin-α gene that are associated with susceptibility to myocardial infarction. Nat. Genet..

[90] Manvich DF (2018). The DREADD agonist clozapine N-oxide (CNO) is reverse-metabolized to clozapine and produces clozapine-like interoceptive stimulus effects in rats and mice. Sci. Rep..

[91] Gomez JL (2017). Chemogenetics revealed: DREADD occupancy and activation via converted clozapine. Science.

[92] Jendryka M (2019). Pharmacokinetic and pharmacodynamic actions of clozapine-N-oxide, clozapine, and compound 21 in DREADD-based chemogenetics in mice. Sci. Rep..

[93] Jun JJ (2017). Fully integrated silicon probes for high-density recording of neural activity. Nature.

[94] Steinmetz NA (2021). Neuropixels 2.0: a miniaturized high-density probe for stable, long-term brain recordings. Science.

[95] Song F, Chan GMA, Wei Y (2020). Flexible experimental designs for valid single-cell RNA-sequencing experiments allowing batch effects correction. Nat. Commun..

[96] Venables, W. N. & Ripley, B. D. Modern applied statistics with S.-PLUS. Springer Science & Business Media (2013).

[97] Benjamini Y, Hochberg Y (1995). Controlling the false discovery rate: a practical and powerful approach to multiple testing. J. R. Stat. Soc..

[98] Hotelling H (1953). New light on the correlation coefficient and its transforms. J. R. Stat. Soc. Ser. B Stat. Methodol..

[99] Storey JD, Tibshirani R (2003). Statistical significance for genomewide studies. Proc. Natl Acad. Sci. USA.

[100] Bates D, Mächler M, Bolker B, Walker S (2015). Fitting linear mixed-effects models using lme4. J. Stat. Softw., Artic..

[101] Hothorn T, Bretz F, Westfall P (2008). Simultaneous inference in general parametric models. Biom. J..

[102] Martín Fernández JA, Daunis i Estadella J, Mateu i Figueras G (2015). On the interpretation of differences between groups for compositional data. Stat. Oper. Res. Trans..

