# Supplementary material for: Cerebellar contributions to a brainwide network for flexible behavior in mice
Source: Commun Biol. 2023 Jun 5;6:605. doi: 10.1038/s42003-023-04920-0 (PMC10241932; doi:10.1038/s42003-023-04920-0)
Supplement: Supplementary file 1 — Supplementary Information [file 42003_2023_4920_MOESM1_ESM.pdf]

Supplementary Information for

Cerebellar contributions to a brainwide network for flexible behavior in mice

Jessica L. Verpeut[1**], Silke Bergeler[1,2,3], Mikhail Kislin[1], F. William Townes[4], Ugne Klibaite[5], Zahra M. Dhanerawala[1], Austin Hoag[1], Sanjeev Janarthanan[1], Caroline Jung[1], Junuk Lee[1], Thomas J. Pisano[1], Kelly M. Seagraves[1], Joshua W. Shaevitz[2,3], Samuel S.-H. Wang[1**]

**Corresponding authors

Correspondence: Samuel S.-H. Wang
sswang@princeton.edu

Jessica Verpeut
jverpeut@asu.edu

This PDF file includes:

Supplementary Figures 1 to 20
Supplementary Tables 1 to 3

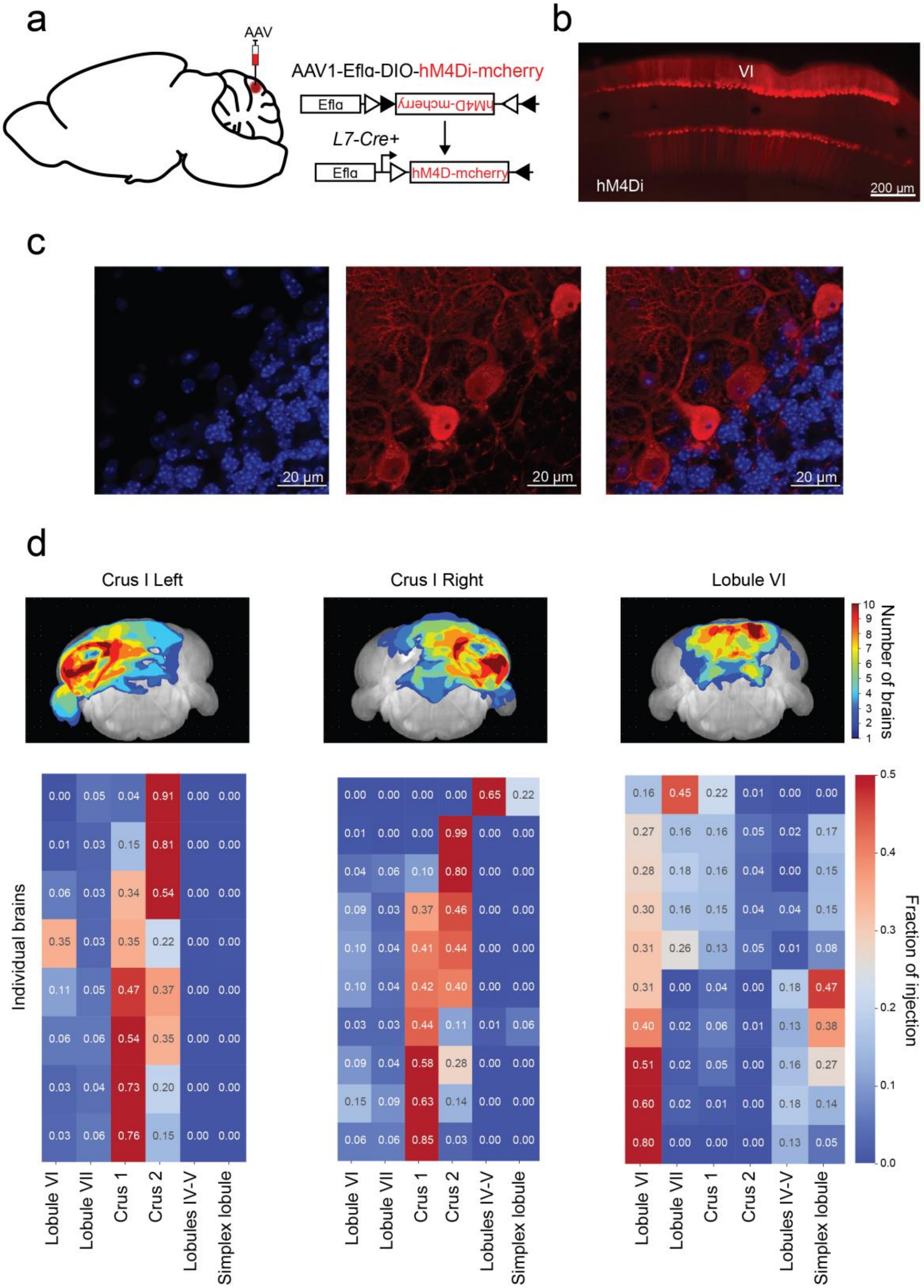
a
AAV
AAV1-Eflα-DIO-hM4Di-mcherry
Eflα
hM4D-mcherry
L7-Cre+
Eflα
hM4D-mcherry
b
VI
hM4Di
200 µm
c
20 µm
20 µm
20 µm
d
Crus I Left
Crus I Right
Lobule VI
Number of brains
10
9
8
7
6
5
4
3
2
1
Individual brains
0.00 0.05 0.04 0.91 0.00 0.00
0.01 0.03 0.15 0.81 0.00 0.00
0.06 0.03 0.34 0.54 0.00 0.00
0.35 0.03 0.35 0.22 0.00 0.00
0.11 0.05 0.47 0.37 0.00 0.00
0.06 0.06 0.54 0.35 0.00 0.00
0.03 0.04 0.73 0.20 0.00 0.00
0.03 0.06 0.76 0.15 0.00 0.00
0.00 0.00 0.00 0.00 0.65 0.22
0.01 0.00 0.00 0.99 0.00 0.00
0.04 0.06 0.10 0.80 0.00 0.00
0.09 0.03 0.37 0.46 0.00 0.00
0.10 0.04 0.41 0.44 0.00 0.00
0.10 0.04 0.42 0.40 0.00 0.00
0.03 0.03 0.44 0.11 0.01 0.06
0.09 0.04 0.58 0.28 0.00 0.00
0.15 0.09 0.63 0.14 0.00 0.00
0.06 0.06 0.85 0.03 0.00 0.00
0.16 0.45 0.22 0.01 0.00 0.00
0.27 0.16 0.16 0.05 0.02 0.17
0.28 0.18 0.16 0.04 0.00 0.15
0.30 0.16 0.15 0.04 0.04 0.15
0.31 0.26 0.13 0.05 0.01 0.08
0.31 0.00 0.04 0.00 0.18 0.47
0.40 0.02 0.06 0.01 0.13 0.38
0.51 0.02 0.05 0.00 0.16 0.27
0.60 0.02 0.01 0.00 0.18 0.14
0.80 0.00 0.00 0.00 0.13 0.05
Fraction of injection
0.5
0.4
0.3
0.2
0.1
0.0
Lobule VI
Lobule VII
Crus 1
Crus 2
Lobules IV-V
Simplex lobule

Supplementary Figure 1. Selective expression of mCherry in Purkinje cells of the cerebellum. (a) AAV carrying hM4Di fused to mCherry was injected into lobule VI or crus I of C57BL/6J mice along with AAV-L7-cre virus to allow for Purkinje cell-specific expression. (b) Sagittal section showing fluorescent expression (epifluorescent images at 40x) revealed by anti-RFP immunohistochemistry 3 weeks after injection in lobule VI. (c) Immunostaining confocal fluorescent images (63x) of lobule VI reveal hM4Di-mCherry expression in Purkinje cells (red). (d) Examples of whole-brain lightsheet averaged injection volumes calculated to the volume of the entire lobule based on immunostaining of mCherry in lobule VI (n=10), crus I left (n=10), crus I right (n=10), bilateral crus I (n=7). Calculated subset of individual brain volumes of the DREADD fraction injected per cerebellar lobule of interest.

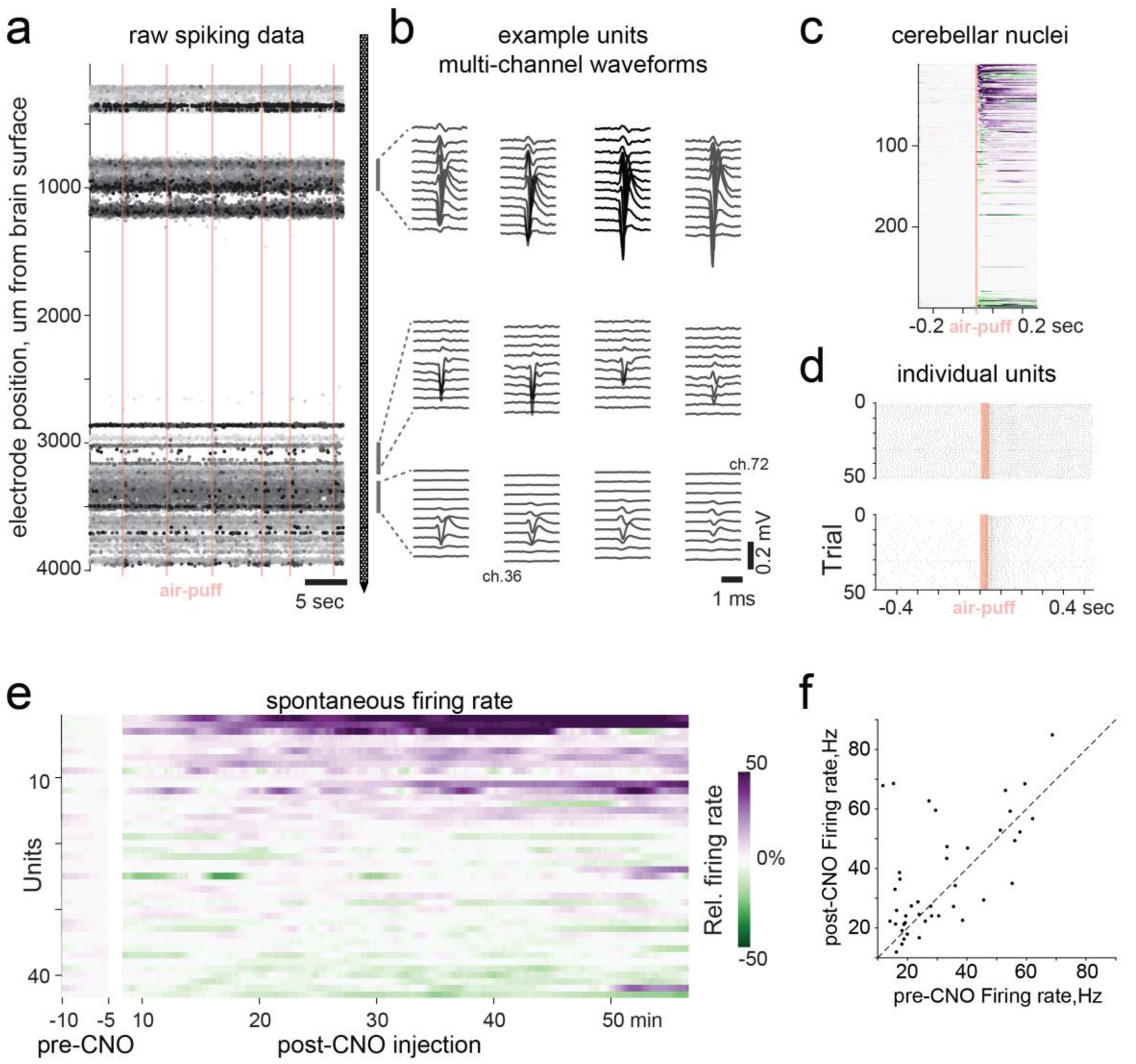

Supplementary Figure 2. Neuropixels recordings of DREADD-expression in deep nuclear cells. (a) Raw spiking data on all 384 electrodes of the probe placed in the cerebellum during a sensory stimulation with air puff (n=5 mice). Stimulus time (40 ms, 1 pulse) is indicated with a red bar. (b) Example units of multi-channel waveforms. (c) Normalized averaged responses to an air-puff stimulation of all sorted units with a mean firing rate of 10 Hz or greater in cerebellar nuclei. (Same color bar as e) (d) Raster of spikes for 50 repetitions of the air-puff stimulation for two example cerebellar nuclei units. Stimulus time (40 ms, 1 pulse) is indicated with a red bar. (e) Spontaneous firing rate of deep cerebellar nuclei neurons (n=43) before and after CNO injection to mice with Purkinje cells expressing DREADD receptors. (f) Relationship between change in pre- and post-CNO firing rate.

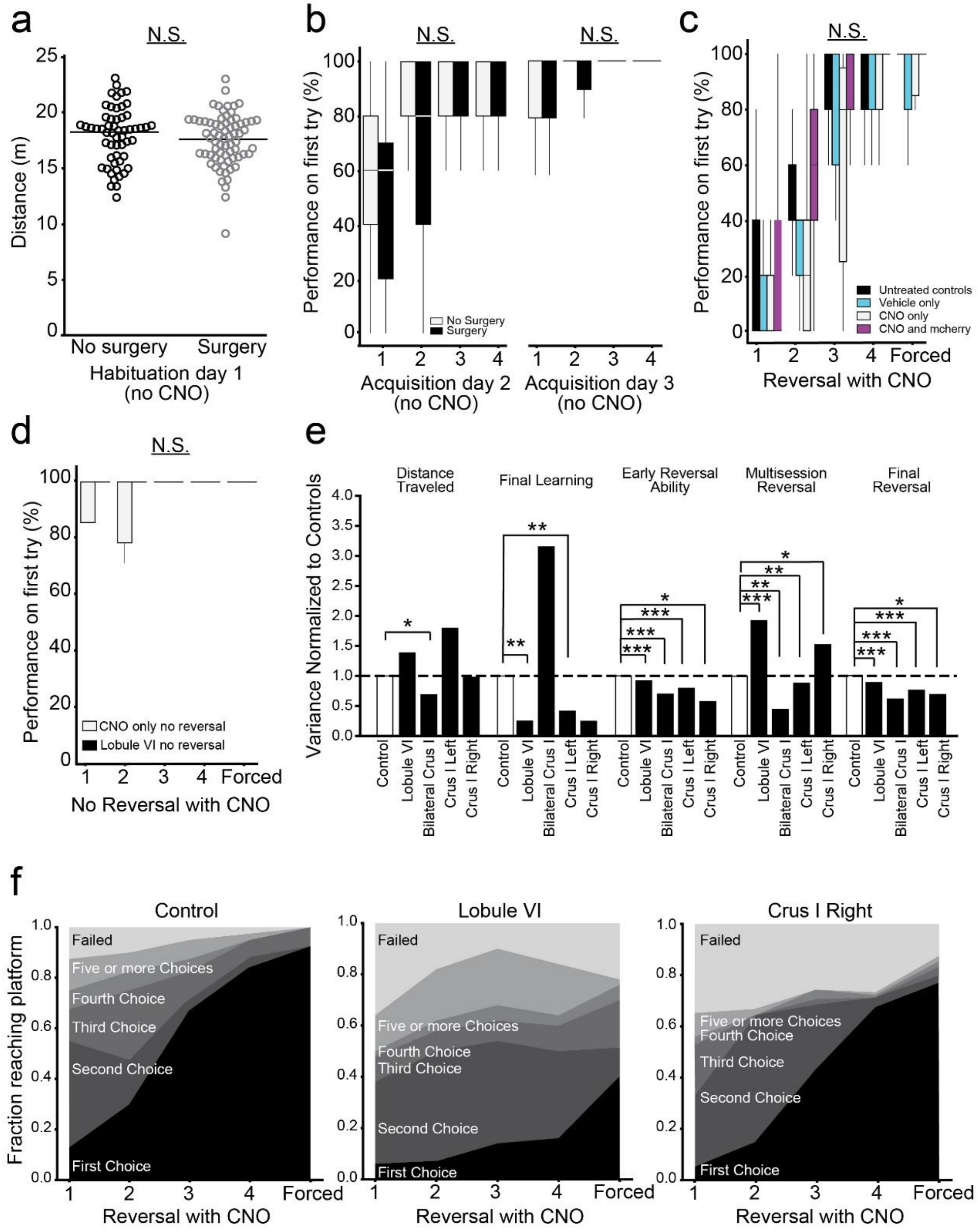

Supplementary Figure 3. Additional Y-maze metrics. (a) Surgery (DREADD experimental conditions and mCherry; n = 102) had no effect on swimming total distance during habituation compared to no surgery (Untreated, Vehicle only, CNO only; n = 101). (b) All animals were taught to swim to one single side of the Y-maze during acquisition sessions. Surgery had no effect on acquisition day 1 or 2. (c) To control for the unintended consequences of CNO, mice were given CNO (n = 22) or vehicle (n = 9) 20 minutes before testing. To control for potential AAV effects, animals received AAV with

mCherry only and CNO (n = 18) during testing. No effects from AAV, CNO, or vehicle were found compared to untreated controls (n = 31). (d) To test if activating the DREADD virus in lobule VI perturbed acquisition, a separate group of lobule VI mice were tested using a third acquisition day (no reversal; n = 10) and compared to a CNO only no reversal group (n = 7). No effects of CNO or DREADD perturbation were found in the no reversal condition. (e) Within-experimental-group variance in distance traveled during learning and reversal for experimental groups and untreated controls. Note that the bilateral crus I group was highly variable and did not reach statistical significance. (f) The number of choices required to find the platform in the Y-maze comparing experimental groups to CNO only control. Comparisons were made using one or two-way repeated measures ANOVA. All box-and-whisker plots show quartiles of the data and whiskers extend to show distribution. * $p < 0.05$, ** $p < 0.01$, *** $p < 0.001$

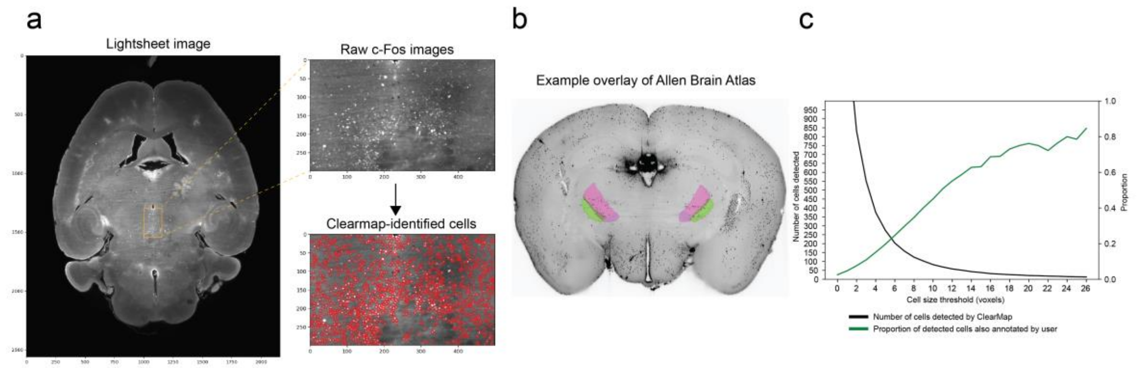

Supplementary Figure 4. Lightsheet image analysis. Example lightsheet image. From each lightsheet digital z-stack, (a) c-Fos is then identified and counted using (a) Clearmap. (b) Example overlay of mouse Allen Brain Atlas showing ventral posteromedial nucleus of the thalamus (pink) and ventral posterolateral nucleus of the thalamus (green). All visualizations were created using Neuroglancer software. (c) Human annotations of 14 brain volumes found 96.72% of cells counted by the human annotators matched ClearMap counted cells.

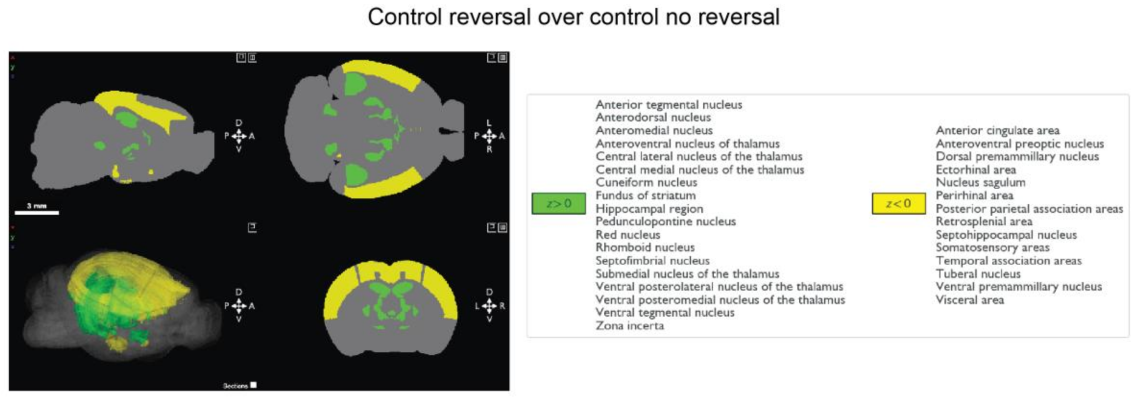

Supplementary Figure 5. Reversal learning alters region-specific activity. Whole-brain Neuroglancer visualization of significantly different regions in CNO only reversal (n=22) compared to no reversal controls (n=7). Reversal resulted in different activated brain regions compared to acquisition. Green: increases, $p < 0.05$, Yellow: decreases, $p < 0.05$

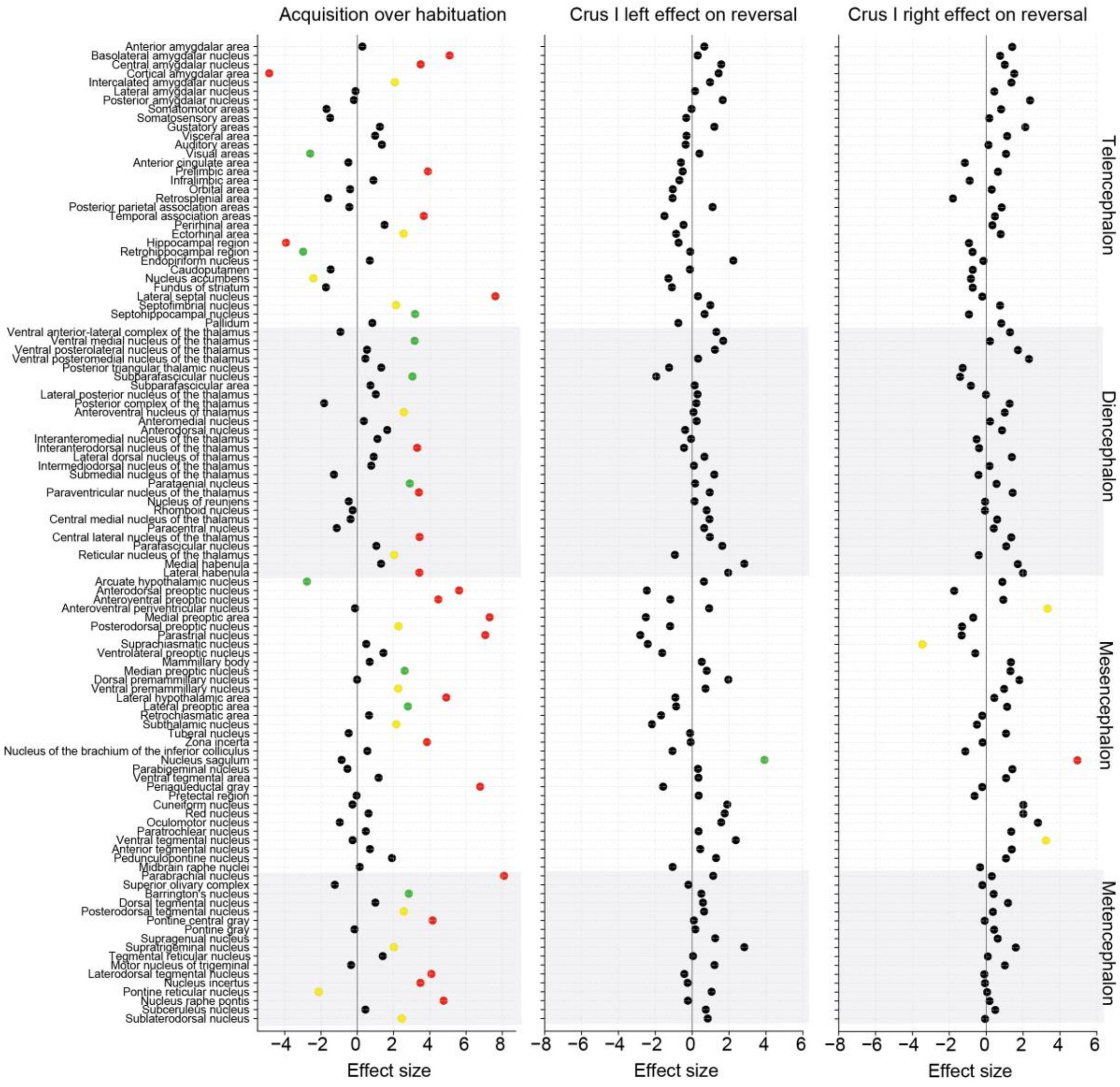

Supplementary Figure 6. Additional comparisons from whole-brain imaging of c-Fos. Comparisons between acquisition only (n=10) and habituation only (n=10) groups are plotted by effect size differences to find structures activated by the acquisition condition. Unilateral crus I left (n=26) and crus I right (n=25) perturbation had little effect on c-Fos cell counts compared to CNO only control, which is consistent with a lack of a behavioral effect during reversal in the water Y-maze task. Comparisons were made using a negative binomial regression.Yellow: $p < 0.05$, Green: $p < 0.01$, Red: $p < 0.001$

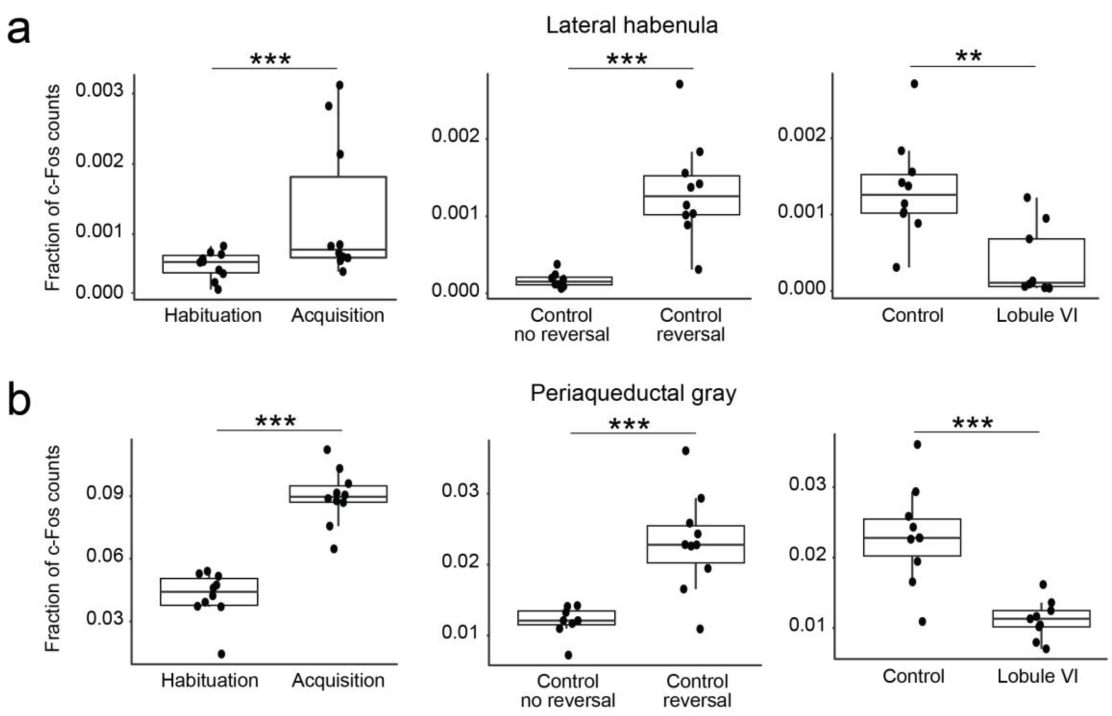

Supplementary Figure 7. c-Fos neural activation in lateral habenula and periaqueductal gray after lightsheet microscopy. (a) Comparison of habituation (n = 10) and acquisition (n = 10) , CNO only controls no reversal (n = 7) and CNO only controls reversal (n = 22), and CNO only controls reversal versus Lobule VI reversal (n = 16). Reversal learning in CNO only controls significantly increased c-Fos in the lateral habenula. Lobule VI perturbation reduced c-Fos counts in the lateral habenula compared to reversal learning. (b) In the periaqueductal gray, Y-maze acquisition and reversal learning in CNO only controls significantly increased c-Fos counts. Lobule VI perturbation reduced c-Fos counts in the periaqueductal gray compared to reversal learning. Comparisons were made using a pairwise t-test. ** $p < 0.01$, *** $p < 0.001$

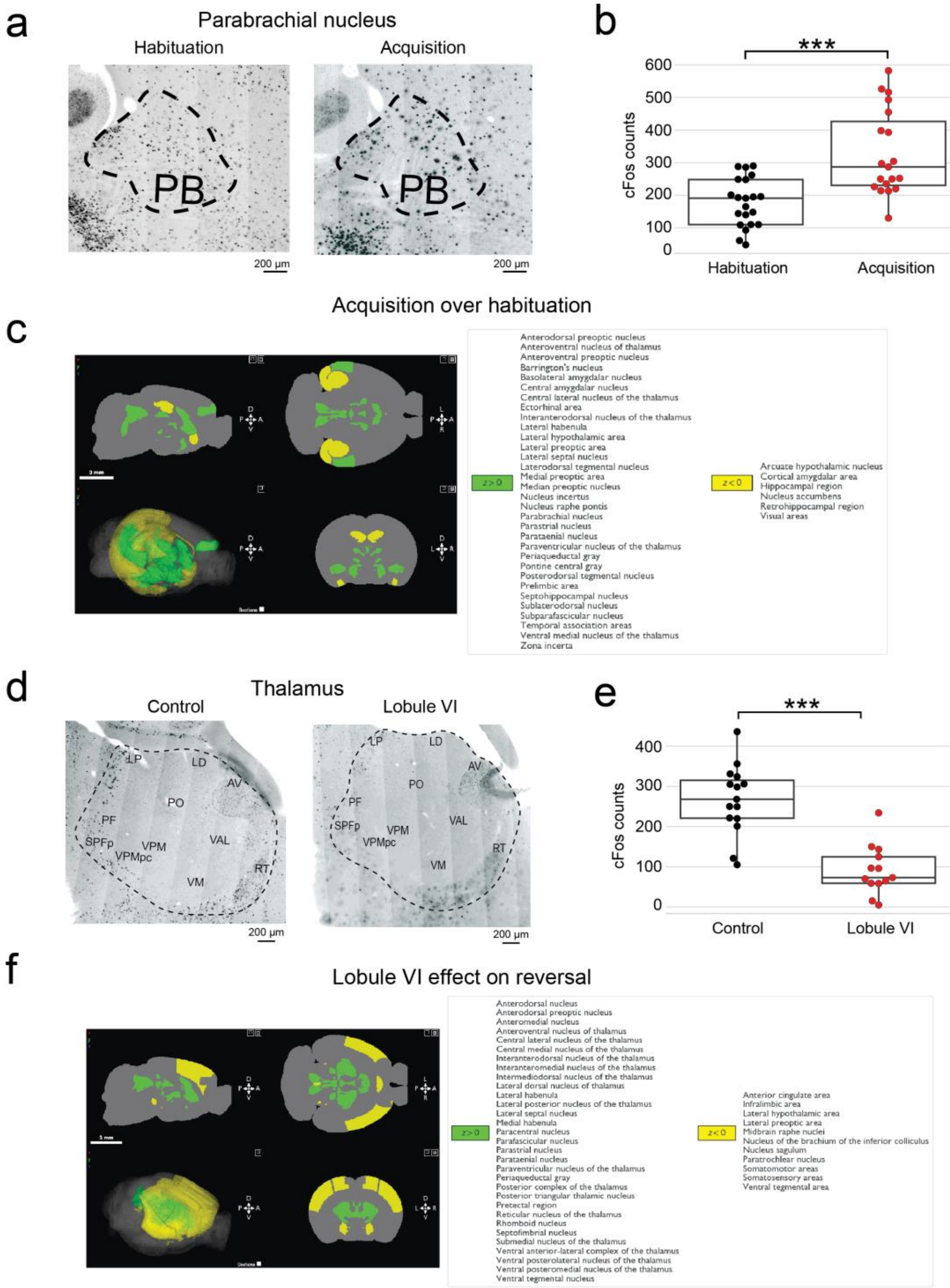

a
Parabrachial nucleus
Habituation
Acquisition
PB
PB
200 µm
200 µm
b
***
600
500
400
300
200
100
0
cFos counts
Habituation
Acquisition
Acquisition over habituation
c
Anterodorsal preoptic nucleus
Anteroventral nucleus of thalamus
Anteroventral preoptic nucleus
Barrington's nucleus
Basolateral amygdalar nucleus
Central amygdalar nucleus
Central lateral nucleus of the thalamus
Ectorhinal area
Interanterodorsal nucleus of the thalamus
Lateral habenula
Lateral hypothalamic area
Lateral preoptic area
Lateral septal nucleus
Laterodorsal tegmental nucleus
Medial preoptic area
Median preoptic nucleus
Nucleus incertus
Nucleus raphe pontis
Parabrachial nucleus
Parastrial nucleus
Parataenial nucleus
Paraventricular nucleus of the thalamus
Periaqueductal gray
Pontine central gray
Posterodorsal tegmental nucleus
Prelimbic area
Septohippocampal nucleus
Sublaterodorsal nucleus
Subparafascicular nucleus
Temporal association areas
Ventral medial nucleus of the thalamus
Zona incerta
z>0
z<0
Arcuate hypothalamic nucleus
Cortical amygdalar area
Hippocampal region
Nucleus accumbens
Retrohippocampal region
Visual areas
d
Thalamus
Control
Lobule VI
LP
LD
AV
PO
PF
SPFp
VPM
VPMpc
VAL
RT
VM
200 µm
200 µm
e
***
400
300
200
100
0
cFos counts
Control
Lobule VI
f
Lobule VI effect on reversal
Anterodorsal nucleus
Anterodorsal preoptic nucleus
Anteromedial nucleus
Anteroventral nucleus of thalamus
Central lateral nucleus of the thalamus
Central medial nucleus of the thalamus
Interanterodorsal nucleus of the thalamus
Interanteromedial nucleus of the thalamus
Intermediodorsal nucleus of the thalamus
Lateral dorsal nucleus of thalamus
Lateral habenula
Lateral posterior nucleus of the thalamus
Lateral septal nucleus
Medial habenula
Paracentral nucleus
Parafascicular nucleus
Parastrial nucleus
Parataenial nucleus
Paraventricular nucleus of the thalamus
Periaqueductal gray
Posterior complex of the thalamus
Posterior triangular thalamic nucleus
Pretectal region
Reticular nucleus of the thalamus
Rhomboid nucleus
Septofimbrial nucleus
Submedial nucleus of the thalamus
Ventral anterior-lateral complex of the thalamus
Ventral posterolateral nucleus of the thalamus
Ventral posteromedial nucleus of the thalamus
Ventral tegmental nucleus
z>0
z<0
Anterior cingulate area
Infralimbic area
Lateral hypothalamic area
Lateral preoptic area
Midbrain raphe nuclei
Nucleus of the brachium of the inferior colliculus
Nucleus sagulum
Paratrochlear nucleus
Somatomotor areas
Somatosensory areas
Ventral tegmental area

Supplementary Figure 8. Confirmation using conventional histology that acquisition and lobule VI perturbation activate a unique set of brain regions in the water Y-maze task. (a) Conventional immunohistochemical staining of c-Fos in the parabrachial nucleus (PB) in habituation only (n = 6) versus acquisition only (n = 6) groups. (b) Acquisition only mice had increased c-Fos staining in PB (each data point represents counts per slice). Scale bar 200 μm (c) Whole-brain Neuroglancer visualization of significantly different regions in acquisition circuits. (d) Conventional immunohistochemical staining of c-Fos in the thalamus of Lobule VI (n = 6) compared to CNO only (n = 6). Lateral posterior nucleus (LP), lateral dorsal nucleus (LD), anteroventral nucleus (AV), posterior complex (PO), ventral anterior-lateral complex (VAL), ventral medial nucleus (VM), reticular nucleus (RT), ventral posteromedial nucleus (VPM), ventral posteromedial nucleus parvicellular part (VPMpc), subparafascicular parvicellular part (SPFp), parafascicular nucleus (PF). (e) Lobule VI mice had significantly less c-Fos in thalamic regions compared to CNO only (each data point represents counts per slice). (f) Whole-brain Neuroglancer visualization of significantly different regions in lobule VI circuits. Green: increases, $p < 0.05$, Yellow: decreases, $p < 0.05$, *** $p < 0.001$

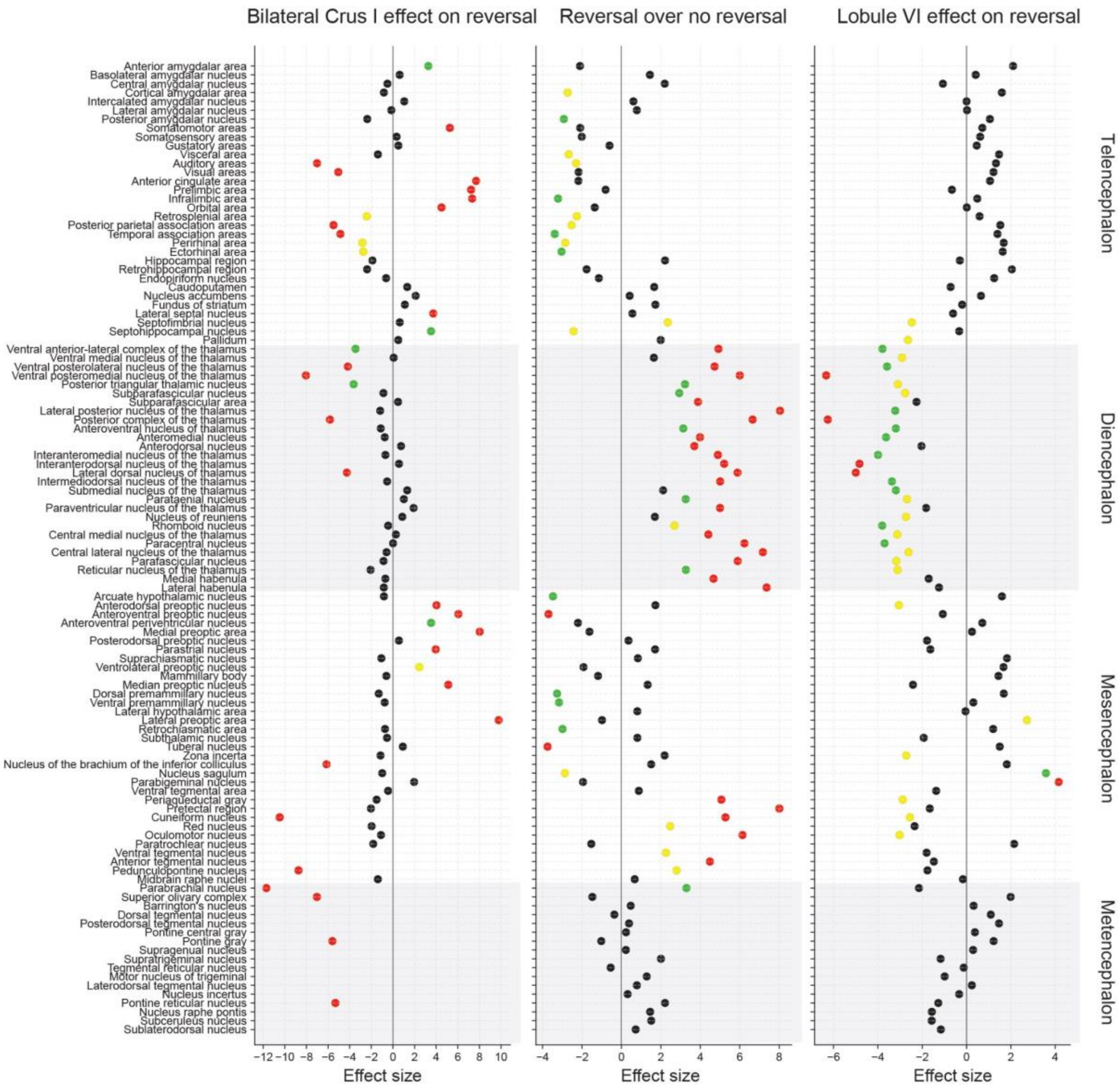

Supplementary Figure 9. Brain-wide association study of activated c-Fos expression with distance swum as a variable. Statistically-significant ($p < 0.05$) bilateral crus I (n=7), CNO only (n=22) lobule VI (n=16) structures compared to CNO only controls. By adding distance swum as a variable in our analysis we find most structures remain significant suggesting that swimming is a separate neural network to Y-maze reversal. Yellow: $p < 0.05$, Green: $p < 0.01$, Red: $p < 0.001$

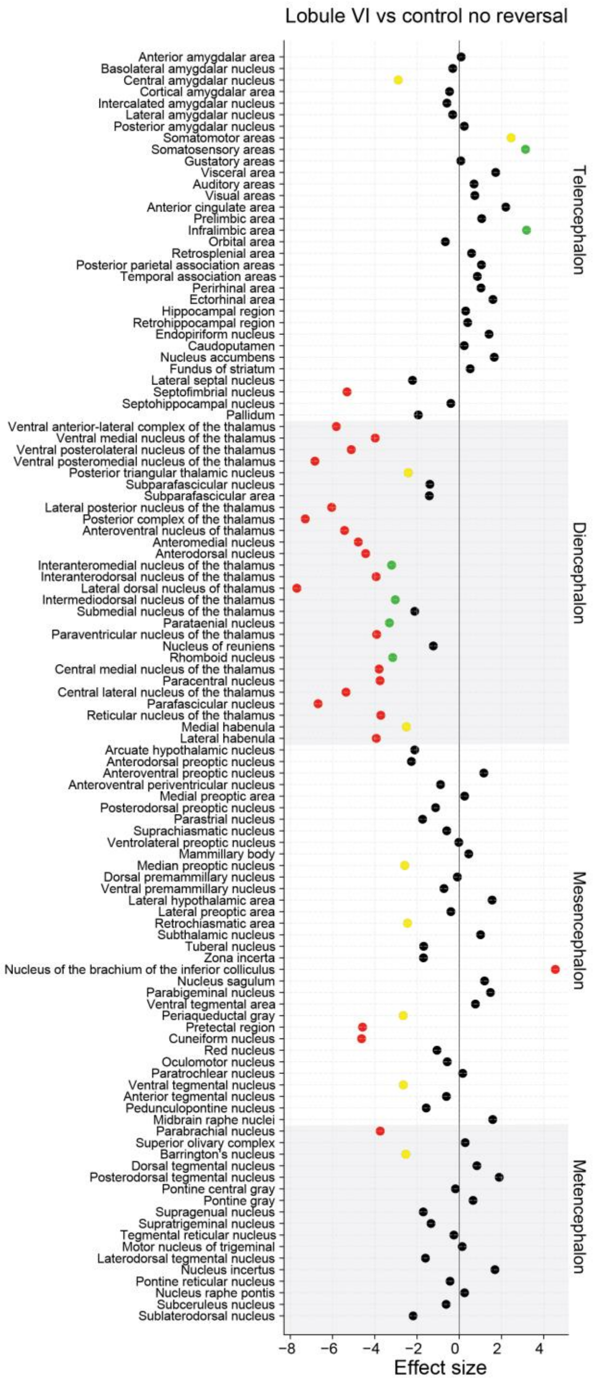

Supplementary Figure 10. Comparison of lobule VI reversal to lobule VI no reversal c-Fos structures. Statistically-significant ($p < 0.05$) lobule VI reversal (n=16) versus lobule VI control no reversal (n=10). Lobule VI perturbation modulated regions necessary for reversal learning, including thalamus. Comparisons were made using a negative binomial regression. Yellow: * $p < 0.05$, Green: ** $p < 0.01$, Red: *** $p < 0.001$

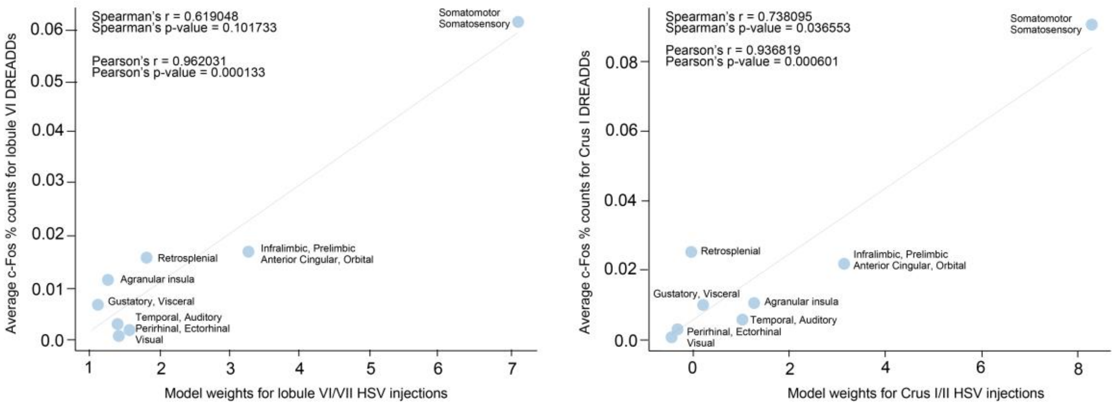

Supplementary Figure 11. Relationship between HSV viral tracing and c-Fos labeled cells. Correlations between lobule VI (n=16) and crus I (n=58) c-Fos (percent of all cells) and viral tracing using herpes simplex virus (HSV-H129) demonstrate strong relationships with neocortical structures. Data from Pisano et al., 2021.

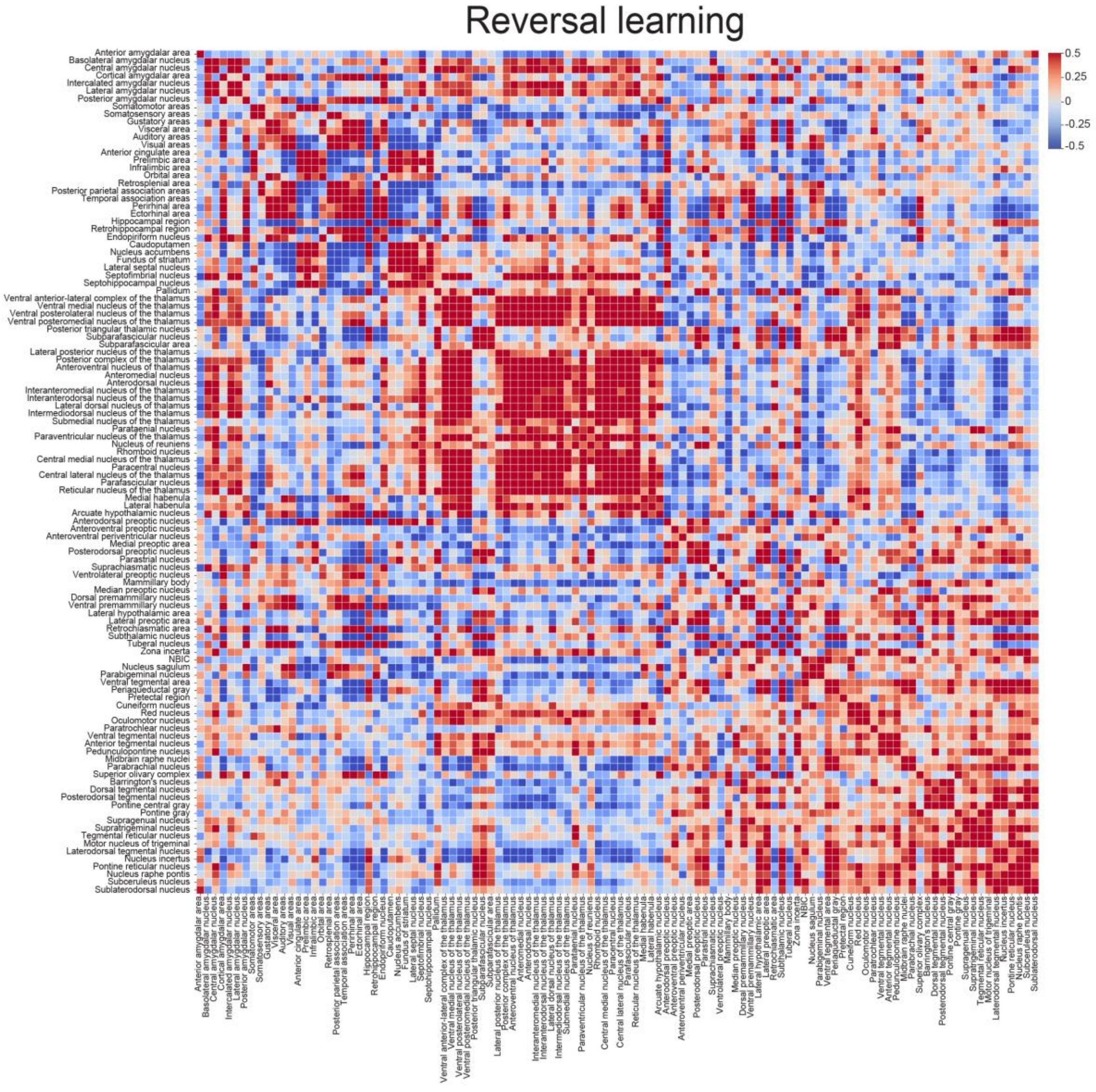

Supplementary Figure 12. Reversal learning correlation matrix. Inter-region connections for c-Fos expression in reversal learning. Strength of correlation reflected in scale bar (Spearman’s p).

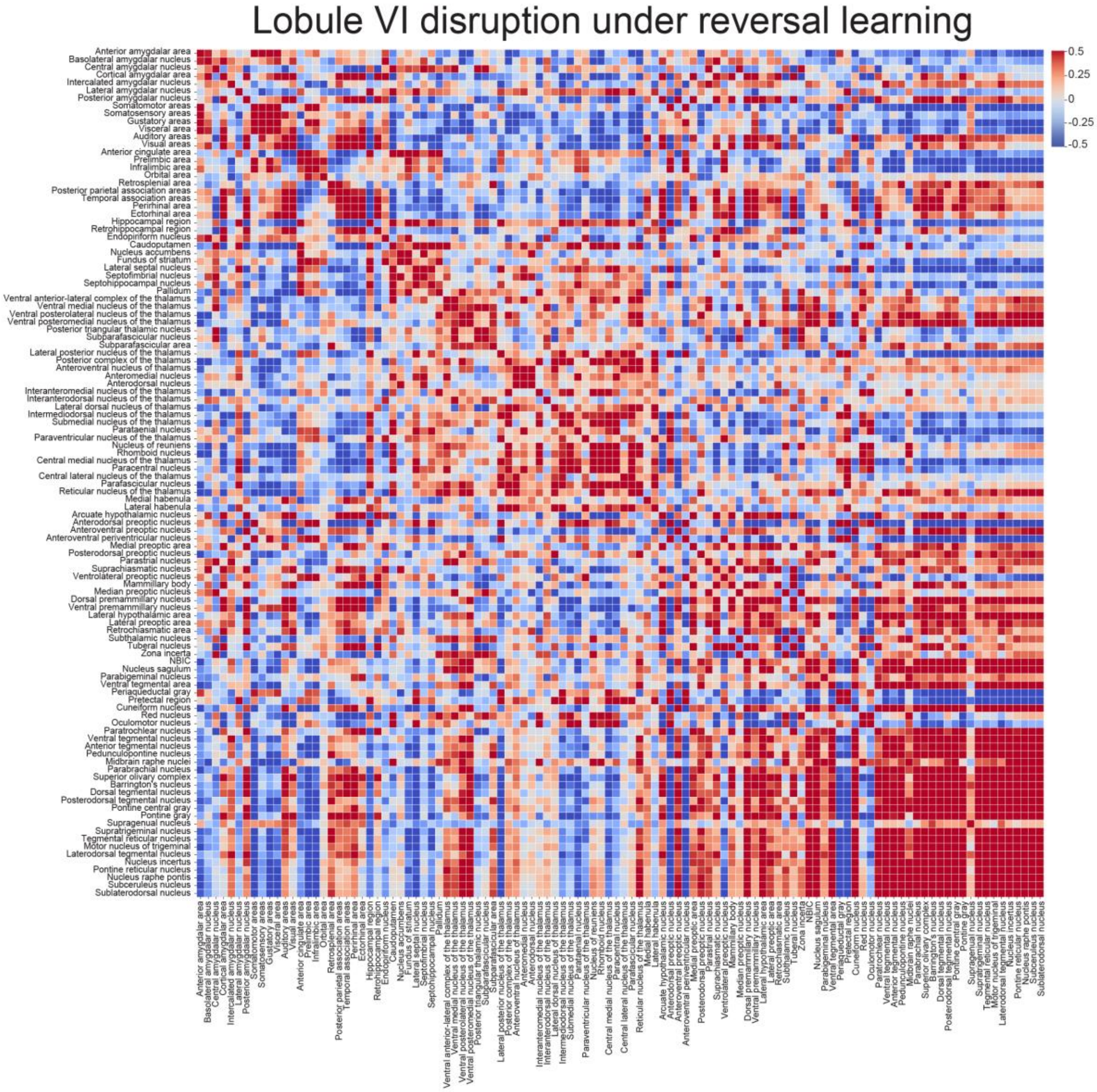

Supplementary Figure 13. Lobule VI disruption in Y-maze reversal. Inter-region connections for c-Fos expression in lobule VI disruption. Strength of correlation reflected in scale bar (Spearman's p).

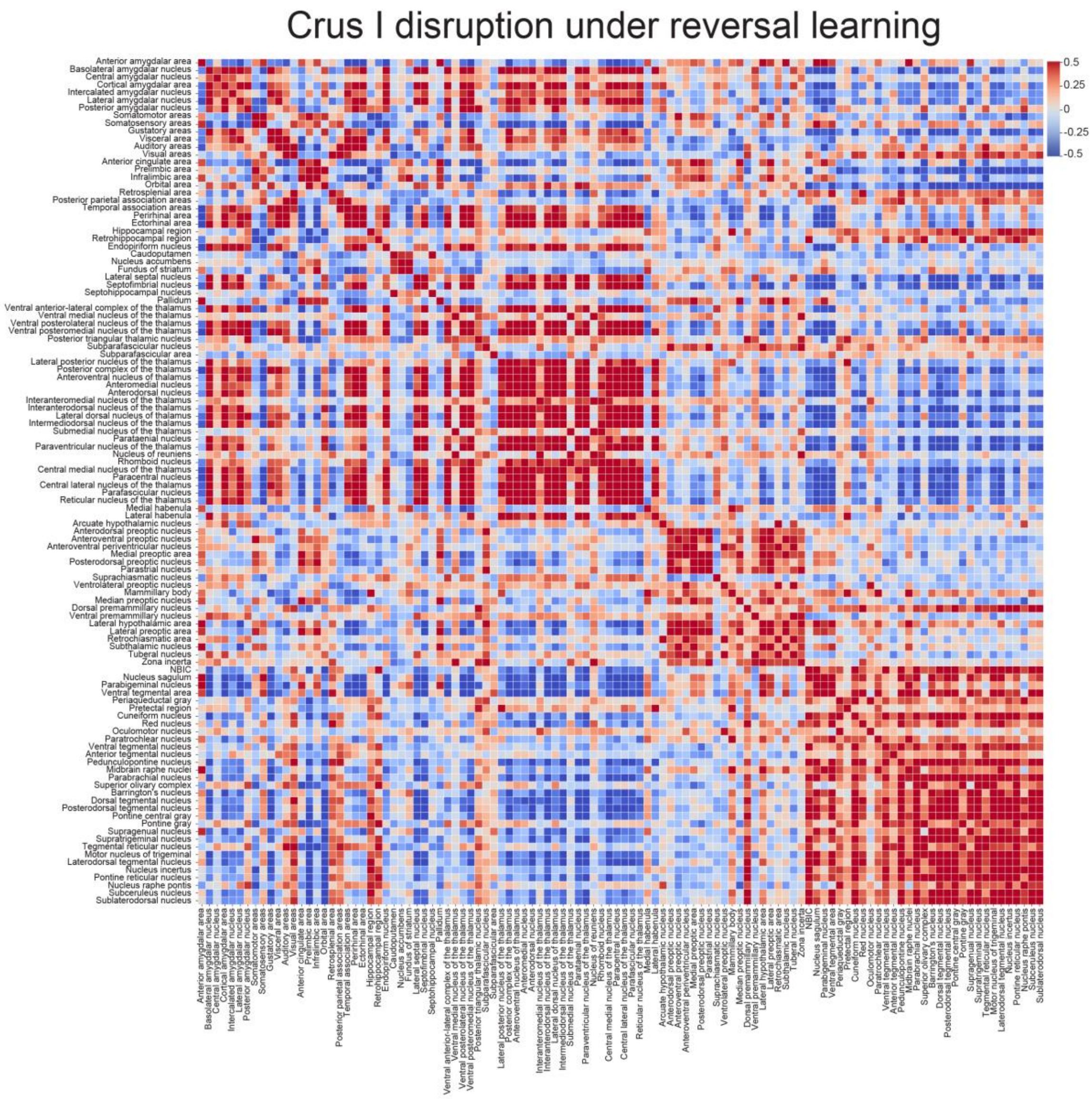

Supplementary Figure 14. Crus I disruption in Y-maze reversal. Inter-region connections for c-Fos expression in crus I disruption. Strength of correlation reflected in scale bar (Spearman's p).

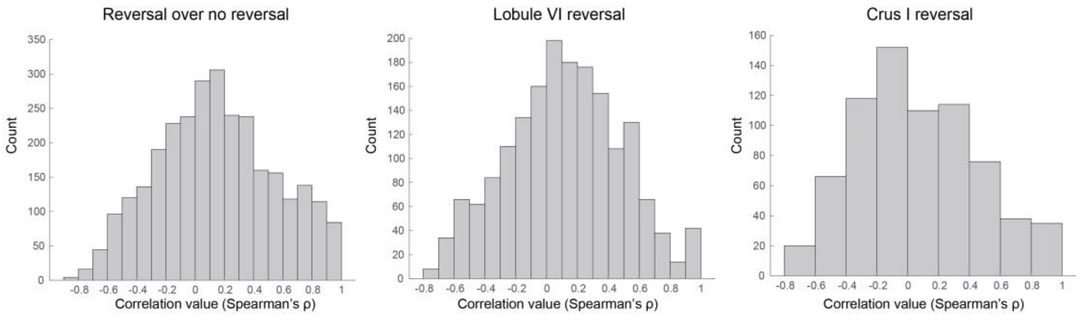

Supplementary Figure 15. Histogram analysis of inter-region correlations. Correlation (Spearman's ρ) histograms for significant regions found in brainwide comparisons of groups at $p < 0.05$ for reversal (CNO only, n = 22), lobule VI disruption (n = 16), and combined crus I disruption (n = 58).

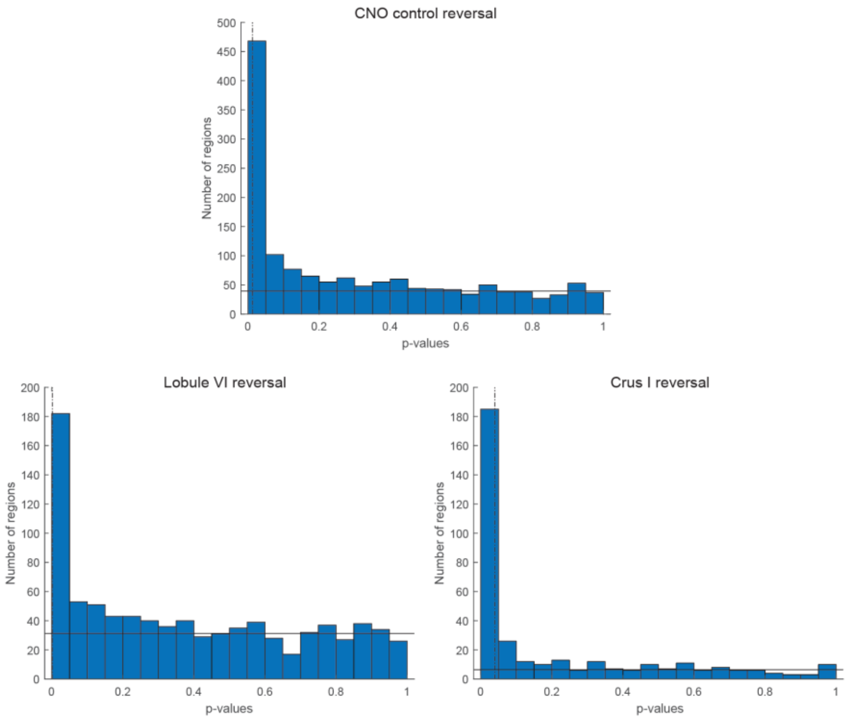

Supplementary Figure 16. Within experimental group histograms of p-values. Histograms for all p-values analyzing inter-region correlations of reversal (CNO only, n = 22), lobule VI disruption (n = 16), and combined crus I disruption (n = 58). Horizontal line is the estimated null distribution.

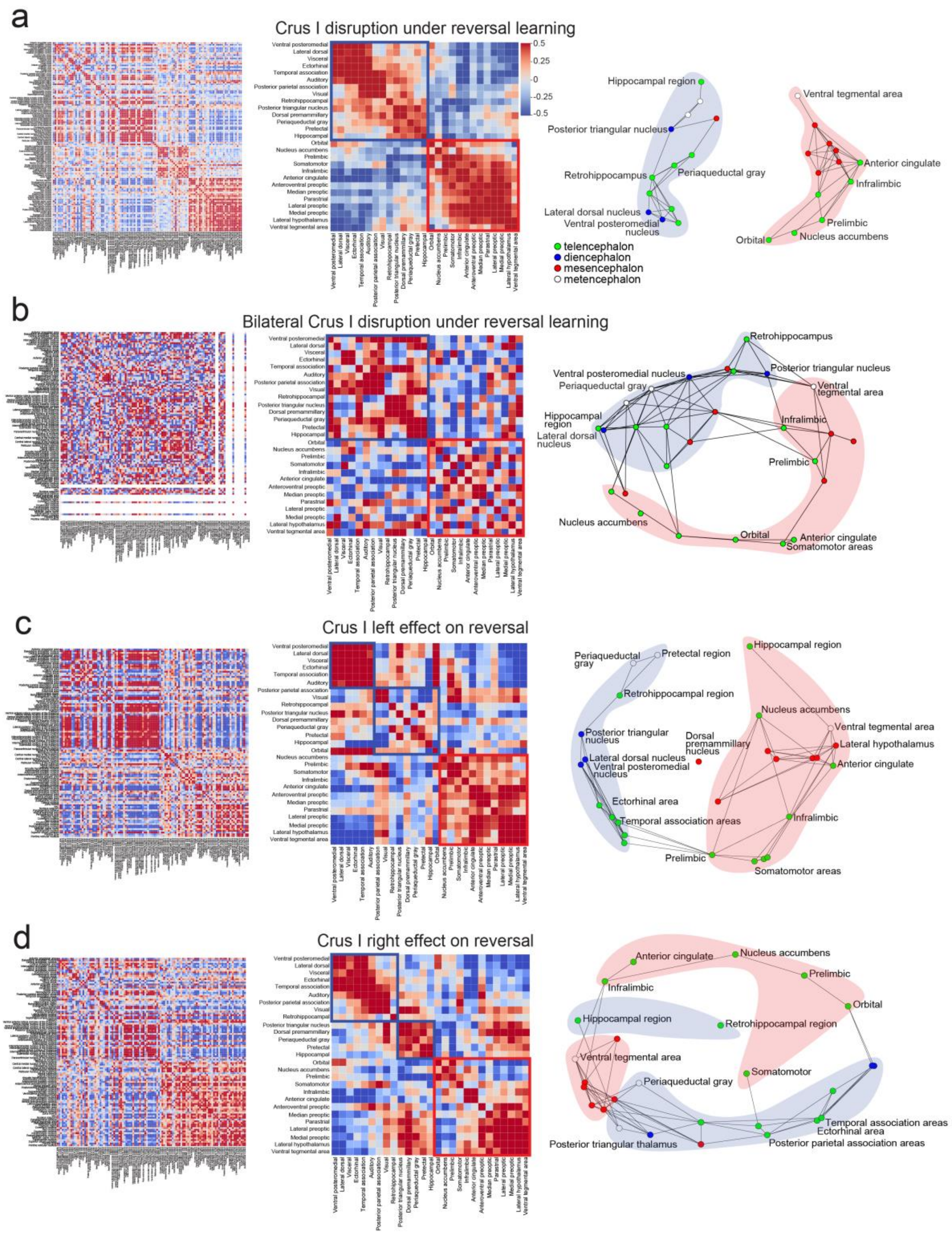
a
Crus I disruption under reversal learning
0.5
0.25
0
-0.25
-0.5
Hippocampal region
Posterior triangular nucleus
Retrohippocampus
Periaqueductal gray
Lateral dorsal nucleus
Ventral posteromedial nucleus
Ventral tegmental area
Anterior cingulate
Infralimbic
Prelimbic
Orbital
Nucleus accumbens
telencephalon
diencephalon
mesencephalon
metencephalon
b
Bilateral Crus I disruption under reversal learning
Retrohippocampus
Ventral posteromedial nucleus
Posterior triangular nucleus
Periaqueductal gray
Ventral tegmental area
Hippocampal region
Lateral dorsal nucleus
Infralimbic
Prelimbic
Nucleus accumbens
Orbital
Anterior cingulate
Somatomotor areas
c
Crus I left effect on reversal
Periaqueductal gray
Pretectal region
Hippocampal region
Retrohippocampal region
Nucleus accumbens
Posterior triangular nucleus
Dorsal premammillary nucleus
Ventral tegmental area
Lateral hypothalamus
Lateral dorsal nucleus
Ventral posteromedial nucleus
Anterior cingulate
Ectorhinal area
Temporal association areas
Infralimbic
Prelimbic
Somatomotor areas
d
Crus I right effect on reversal
Anterior cingulate
Nucleus accumbens
Infralimbic
Prelimbic
Orbital
Hippocampal region
Retrohippocampal region
Ventral tegmental area
Somatomotor
Periaqueductal gray
Temporal association areas
Ectorhinal area
Posterior triangular thalamus
Posterior parietal association areas

Supplementary Figure 17. Crus I influence on water Y-maze reversal network. Correlation matrices of all brain regions within (a) crus I disruption (all crus groups combined, n=18 mice), (b) bilateral crus I disruption (n=7 mice), (c) crus I left disruption (n=26 mice), (d) crus I right disruption (n=27 mice). Correlation matrices of significant brain regions ($p < 0.05$) showing inter-region connections for c-Fos expression with (a) crus I disruption (all crus groups combined), (b) bilateral crus I disruption, (c) crus I left disruption, (d) crus I right disruption. Strength of correlation reflected in scale bar (Spearman's rho). Network graphs were generated to visualize relationships between major brain structures (mesencephalon: red, telencephalon: green, metencephalon: white, diencephalon: blue) based on correlations in (a) crus I disruption (all crus groups combined), (b) bilateral crus I disruption, (c) crus I left disruption, (d) crus I right disruption. Abbreviation: Nucleus of the brachium of the inferior colliculus (NBIC).

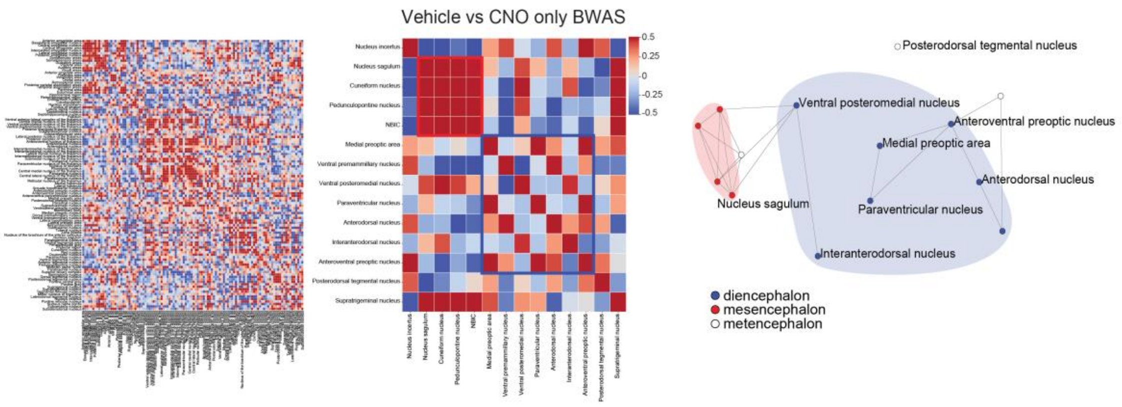

Supplementary Figure 18. BWAS comparison of Vehicle only and CNO only reversal. Correlation matrices of all brain regions, significant brain regions, network relationships between Vehicle only (n=9) and CNO only (n=22) control group. Network graphs were generated to visualize relationships between major brain structures (mesencephalon: red, metencephalon: white, diencephalon: blue). Abbreviation: Nucleus of the brachium of the inferior colliculus (NBIC).

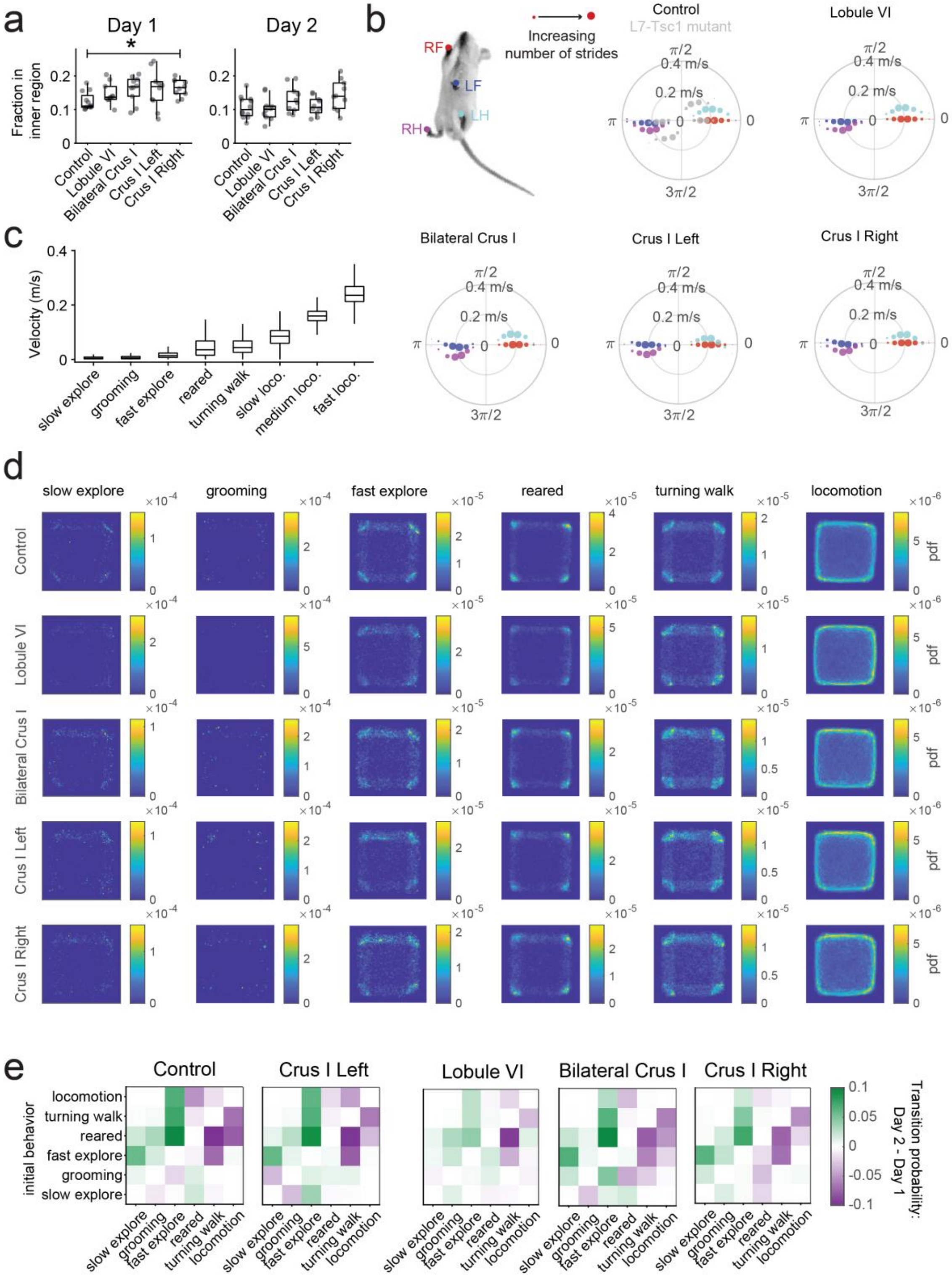

a
Day 1
Day 2
Fraction in inner region
Control
Lobule VI
Bilateral Crus I
Crus I Left
Crus I Right
b
RF
LF
LH
RH
Increasing number of strides
Control
L7-Tsc1 mutant
Lobule VI
Bilateral Crus I
Crus I Left
Crus I Right
0.4 m/s
0.2 m/s
c
Velocity (m/s)
slow explore
grooming
fast explore
reared
turning walk
slow loco.
medium loco.
fast loco.
d
slow explore
grooming
fast explore
reared
turning walk
locomotion
pdf
e
Control
Crus I Left
Lobule VI
Bilateral Crus I
Crus I Right
initial behavior
locomotion
turning walk
reared
fast explore
grooming
slow explore
Transition probability: Day 2 - Day 1

Supplementary Figure 19. Lobule-specific alterations of spontaneous behavior in open field. (a) Fraction of time spent in the inner region of the arena of control (CNO only; n = 12), Lobule VI (n = 10), bilateral crus I (n = 10), crus I left (n = 10), crus I right (n = 10). Comparisons of the groups within each day to CNO only control were made using a Kruskal-Wallis test, followed up by pairwise comparisons using Wilcoxon rank sum exact tests with Benjamini-Hochberg correction. (b) Limb coordination during locomotion is not impaired by cerebellar perturbations. The maximal values of the paw positions in an animal-centered coordinate system were assumed at similar phases during single strides of locomotion. For comparison altered limb coordination in *L7-Tsc1* mutant mice (n = 9) compared to controls (n = 60) is shown from previously published experiments (Klibaite et al. 2022). (c) Centroid velocities during different behaviors. (d) The spatial distributions of occurrences of the different behaviors in the open field arena are similar for each experimental group compared to CNO only control. Data from the recording on day 1 was considered. (e) Lobule VI-perturbed CNO only control mice showed the smallest differences in the state transition probabilities across days. * $p < 0.05$

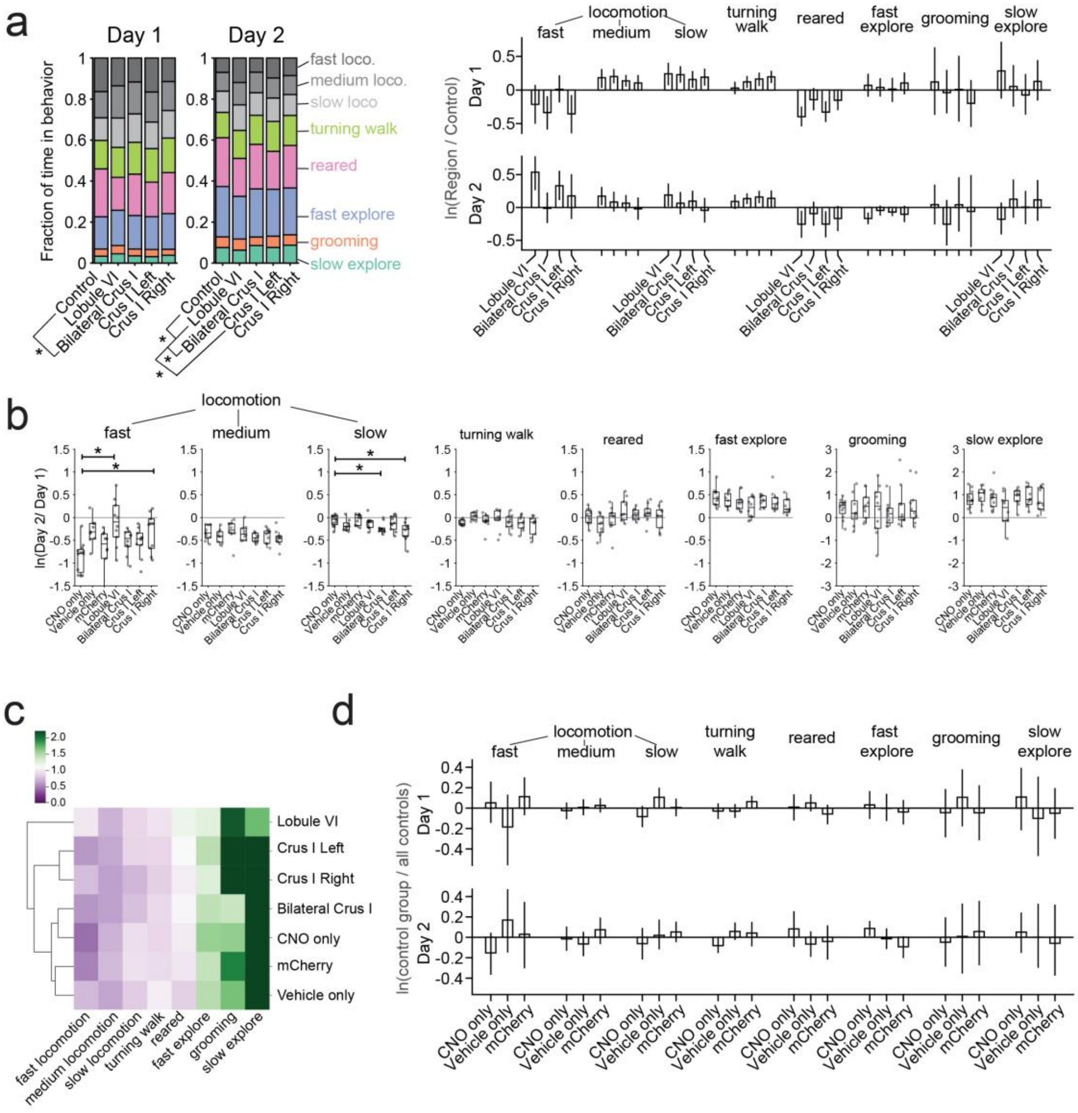

Supplementary Figure 20. Comparison of spontaneous behavior in control groups. (a) Fractions of time spent in each of the eight behaviors of control (CNO only; n = 12), Lobule VI (n = 10), bilateral crus I (n = 10), crus I left (n = 10), crus I right (n = 10). The fractions of time spent in each of the eight behaviors (compositional means) differ significantly between lobule VI-perturbed mice and the CNO only control group on day 2 (nonparametric multivariate test on ilr-transformed fractions, ssnonpartest from R package npmv, Wilks' Lambda type statistic). This difference can be mainly accounted for by the large amount of time spent in fast locomotion on the second day compared to the control, quantified by the log ratio differences between the cerebellar perturbed groups and the control (error bars indicate bootstrapped 95% confidence intervals, N = 5000, percentile bootstrap) (b) Cerebellar perturbations of lobule VI, crus I right and bilateral crus I had an effect on the habituation of the occupancy in slow and fast locomotion states across days compared to CNO only control (Kruskal-Wallis tests for each behavior, pairwise comparisons using Wilcoxon rank sum exact test, Benjamini-

Hochberg correction). (c) The state occupancies (Day 2/Day 1) for all experimental and control groups show clustering between controls (CNO only, n = 12; mCherry, n = 10; Vehicle only, n = 9) compared to experimental groups. Crus I right and left cluster together and have similar state occupancies. Lobule VI-perturbed mice are the most different from all other groups, emphasizing that the lack of habituation creates an unique phenotype. (d) All control groups show a similar change of behavioral occupancies across days (Kruskal-Wallis tests for each behavior, pairwise comparisons using Wilcoxon rank sum exact test, Benjamini-Hochberg correction). * $p < 0.05$

Supplemental Table 1: Y-maze distance swum metrics (m).

	Habituation only	Acquisition only	Control reversal	Lobule VI reversal	Crus I bilateral reversal	Crus I right reversal	Crus I left reversal	CNO no reversal	Lobule VI no reversal
N	10	10	22	16	7	25	26	7	10
Mean	54.6	23.2	20.2	22.7	25.8	22.3	22.3	17.1	16.9
SD	12.2	6.2	6.0	8.6	7.4	6.8	7.4	4.8	3.6

Supplemental Table 2: Key Resources Table

Reagent type (species) or resource	Designation	Source or reference	Identifiers
strain, strain background	Mouse :C57BL/6J	The Jackson Laboratory, Bar Harbor, ME	Stock#: 00664 |Black6 https://www.jax.org/strain/000664
antibody	secondary donkey anti-rabbit Alexa Fluor 647	ThermoFisher	Cat#A-21449
antibody	rabbit anti-RFP	Rockland Immunochemicals	Cat#:600-401-379
antibody	rabbit anti-Fos	Synaptic Systems	Cat#:226 003
recombinant DNA reagent	AAV1-Eflα-DIO-hM4D(Gi)-mCherry-WPRE-hGHpA;AAV8-Eflα-DIO-mCherry-WPRE-hGHpA	Princeton Vector Core	
recombinant DNA reagent	AAV1-sL7-Cre-HA-WPRE-hGH-pA	Princeton Vector Core	
chemical compound, drug	Clozapine-N-oxide (CNO)	NIMH Chemical Synthesis and Drug Supply Program	Cat# 34233-69-7
chemical compound	ProLong™ Diamond Antifade Mountant	ThermoFisher	Cat# P36961
chemical compound, drug	15% D-mannitol	SIGMA-ALDRICH	Cat# M4125
chemical compound	DPBS	ThermoFisher	Cat#14190136
chemical compound	CM-Dil	ThermoFisher	Cat #C7001
chemical compound, drug	white tempera paint	Artmind, Tempera Paint	Cat#10091773
chemical compound, drug	Rimadyl [carprofen]	Zoetis, Florham Park, NJ	http://www.zoetisus.com
chemical compound, drug	ketamine/xylazine	Met-Vet International/Akorn	RXV CIII (3N)/Cat# 59399-111-50
chemical compound, drug	Euthasol	Met-Vet International/Virbac	RXV CIII (3N)/ RXEUTHASOL
software, algorithm	Illustrator CS	Adobe	
	Excel	Microsoft	
	MATLAB R2020b	MathWorks	
	Python 2.7.14/3.8.6	Python	
	R 4.0.3	R	
	ImageJ	NIH	
	Neuroglancer	(Google WebGL-based viewer)	
	Allen Brain Atlas	(Oh et al., 2014).	http://www.brain-map.org
Resource Availability	Data for this manuscript	Princeton database	https://doi.org/10.34770/c9df-sc15
	Data from Klibaite et al., 2022	Princeton database	https://dataspace.princeton.edu/handle/88435/dsp012j62s793m
	Code	Github	https://github.com/PrincetonUniversity/OF-ymaze-cfos-analysis

Supplemental Table 3: Control and experimental conditions per figure.

Control group	Experimental group	Figure
	Lobule VI	Fig 1, Supplemental Figure 1b-c, Supplemental Figure 2
	Lobule VI, Crus I Left, Crus I Right	Supplemental Figure 1d
CNO only	Lobule VI	Fig 4, Fig 7c, Supplemental Figure 8d-f, Supplemental Figure 13
CNO only	Crus I all	Supplemental Figure 14
CNO only, CNO only no reversal		Fig 3, Supplemental Figure 5, Supplemental Figure 12
CNO only	Lobule VI, Bilateral Crus I, Crus I Left, Crus I Right	Fig 2b-c, Fig 7d-e, Supplemental Figure 3e, Supplemental Figure 19a and c-e, Supplemental Figure 20a
CNO only	Lobule VI, Bilateral Crus I	Fig 2d, Fig 6
CNO only, Acquisition, Habituation	Crus I Left, Crus I Right	Supplemental Figure 6
Lobule VI no reversal	Lobule VI reversal	Supplemental Figure 10
Lobule VI no reversal, CNO only no reversal		Supplemental Figure 3d
CNO only, CNO only no reversal	Lobule VI, Bilateral Crus I	Supplemental Figure 9
CNO only, Vehicle only, CNO and mCherry		Supplemental Figure 20d
CNO only, Vehicle only		Supplemental Figure 18
CNO only, Vehicle only, CNO and mCherry	Lobule VI, Bilateral Crus I, Crus I Left, Crus I Right	Supplemental Figure 20b-c
CNO only, Vehicle only, CNO and mCherry, Untreated only		Supplemental Figure 3c
CNO only	Lobule VI, Crus I Right	Supplemental Figure 3f
Acquisition, Habituation		Supplemental Figure 8a-c
Acquisition, Habituation, CNO only, CNO no reversal	Lobule VI	Supplemental Figure 7
	Crus I all: Bilateral Crus I, Crus I Left, Crus I Right	Supplemental Figure 17
CNO only	Lobule VI, Crus I all	Fig 5, Supplemental Figure 15, Supplemental Figure 16
CNO only	L7Cre; Tsc1$^{flox/flox*}$.Lobule VI, Bilateral Crus I, Crus I Left, Crus I Right	Supplemental Figure 19b
