# Supplementary material for: Cerebellar contributions to a brainwide network for flexible behavior in mice
Source: Commun Biol. 2023 Jun 5;6:605. doi: 10.1038/s42003-023-04920-0 (PMC10241932; doi:10.1038/s42003-023-04920-0)
Supplement: Supplementary file 2 — Description of Additional Supplementary Files [file 42003_2023_4920_MOESM2_ESM.pdf]

Description of Additional Supplementary Files

File name: Supplementary Data 1

Description: Effects of experimental perturbations on c-Fos cell counts. Contrasts include acquisition versus habituation, reversal learning versus acquisition, and lobule VI perturbation via DREADDs against CNO only. For each brain region and contrast, negative binomial (NB) regression was performed with natural log of total counts across all regions as an offset. Estimate: NB slope coefficient for effect of treatment (log-ratio scale), Fold change: exponentiated coefficient (ratio scale), Std. Error: standard error of estimate, z value: estimate divided by standard error (also known as effect size), Pr(>|z|): raw p-value for effect, fdr_adj_pval: p-value adjusted for false discovery rate, status: whether NB fitting routine encountered numerical problems.

File name: Supplementary Data 2

Description: Correlation matrices of significant brain regions (p < crus I 0.05) showing inter-region connections for c-Fos expression with CNO only reversal, lobule VI disruption, crus I disruption (all crus groups combined), crus I left, crus I right, and vehicle only. From this data, heat maps of correlations plus network graphs were generated to visualize relationships between major brain structures.

File name: Supplementary Movie 1

Description: Y-maze task. Example video recording of mouse performing Y-maze acquisition, reversal with CNO only, and lobule VI perturbed mouse attempting reversal.

File name: Supplementary Movie 2

Description: Whole brain lightsheet. Example movie of a cleared brain using iDISCO+ and c-Fos immunohistochemistry.

File name: Supplementary Movie 3

Description: High-resolution digital z-stack scan through a whole mouse brain focused on midbrain structures. Bright white cells are labeled c-Fos cells.

File name: Supplementary Movie 4

Description: Semi-supervised behavioral classification. Example video recording of mouse in open field, zoomed-in version with LEAP labels and corresponding ethogram.
