# Supplementary material for: Cerebellar contributions to a brainwide network for flexible behavior in mice
Source: Commun Biol. 2023 Jun 5;6:605. doi: 10.1038/s42003-023-04920-0 (PMC10241932; doi:10.1038/s42003-023-04920-0)
Supplement: Supplementary file 9 — Reporting Summary [file 42003_2023_4920_MOESM9_ESM.pdf]

nature portfolio

Corresponding author(s): Jessica Verpeut and Samuel S.-H. Wang

Last updated by author(s): 4.25.23

Reporting Summary

Nature Portfolio wishes to improve the reproducibility of the work that we publish. This form provides structure for consistency and transparency in reporting. For further information on Nature Portfolio policies, see our Editorial Policies and the Editorial Policy Checklist.

Statistics

For all statistical analyses, confirm that the following items are present in the figure legend, table legend, main text, or Methods section.

n/a	Confirmed	
☐	☒	The exact sample size (*n*) for each experimental group/condition, given as a discrete number and unit of measurement
☐	☒	A statement on whether measurements were taken from distinct samples or whether the same sample was measured repeatedly
☐	☒	The statistical test(s) used AND whether they are one- or two-sided<br>*Only common tests should be described solely by name; describe more complex techniques in the Methods section.*
☐	☒	A description of all covariates tested
☐	☒	A description of any assumptions or corrections, such as tests of normality and adjustment for multiple comparisons
☐	☒	A full description of the statistical parameters including central tendency (e.g. means) or other basic estimates (e.g. regression coefficient) AND variation (e.g. standard deviation) or associated estimates of uncertainty (e.g. confidence intervals)
☐	☒	For null hypothesis testing, the test statistic (e.g. *F*, *t*, *r*) with confidence intervals, effect sizes, degrees of freedom and *P* value noted<br>*Give P values as exact values whenever suitable.*
☒	☐	For Bayesian analysis, information on the choice of priors and Markov chain Monte Carlo settings
☐	☒	For hierarchical and complex designs, identification of the appropriate level for tests and full reporting of outcomes
☐	☒	Estimates of effect sizes (e.g. Cohen's *d*, Pearson's *r*), indicating how they were calculated

Our web collection on statistics for biologists contains articles on many of the points above.

Software and code

Policy information about availability of computer code

Data collection	n/a
Data analysis	https://github.com/PrincetonUniversity/OF-ymaze-cfos-analysis, https://github.com/PrincetonUniversity/OF-ymaze-cfos-analysis

For manuscripts utilizing custom algorithms or software that are central to the research but not yet described in published literature, software must be made available to editors and reviewers. We strongly encourage code deposition in a community repository (e.g. GitHub). See the Nature Portfolio guidelines for submitting code & software for further information.

Data

Policy information about availability of data

All manuscripts must include a data availability statement. This statement should provide the following information, where applicable:

- Accession codes, unique identifiers, or web links for publicly available datasets
- A description of any restrictions on data availability
- For clinical datasets or third party data, please ensure that the statement adheres to our policy

The dataset is available at Princeton data DOI: https://doi.org/10.34770/c9df-sc15, https://brainmaps.princeton.edu/2022/01/verpeut-et-al-data-exploration-links/, https://dataspace.princeton.edu/handle/88435/dsp012j62s793m

All experimental and analysis code is available here: https://github.com/PrincetonUniversity/OF-ymaze-cfos-analysis

Human research participants

Policy information about studies involving human research participants and Sex and Gender in Research.

Reporting on sex and gender	n/a
Population characteristics	n/a
Recruitment	n/a
Ethics oversight	n/a

Note that full information on the approval of the study protocol must also be provided in the manuscript.

Field-specific reporting

Please select the one below that is the best fit for your research. If you are not sure, read the appropriate sections before making your selection.

[x] Life sciences [ ] Behavioural & social sciences [ ] Ecological, evolutionary & environmental sciences

For a reference copy of the document with all sections, see nature.com/documents/nr-reporting-summary-flat.pdf

Life sciences study design

All studies must disclose on these points even when the disclosure is negative.

Sample size	Sample size/animal numbers were estimated by power analysis using an R script that takes pre-specified effect size, type I (α= 0.05) and II errors (1-β = 0.8) as input arguments.
Data exclusions	No data was excluded from analysis
Replication	In each comparison, we processed brains from all control and treatment animals as a single batch using the same tissue preparation and imaging conditions whenever possible. In the few cases where we needed multiple batches, we adjusted for confounding batch effects by including indicator variables for batches as covariates in regression models.
Randomization	All animals were randomized at the time of surgery.
Blinding	All animals were randomized at the time of surgery. Individuals testing behavior were not aware of the experimental groups by using a separate code to label animals. Data was uncoded for final figure creation and data analysis. All data from lightsheet analysis was given to a statistician for analysis who was unaware of the experimental groups. Behavior analysis using machine learning was automated to prevent bias.

Reporting for specific materials, systems and methods

We require information from authors about some types of materials, experimental systems and methods used in many studies. Here, indicate whether each material, system or method listed is relevant to your study. If you are not sure if a list item applies to your research, read the appropriate section before selecting a response.

Materials & experimental systems

n/a	Involved in the study	
[ ]	[x]	Antibodies
[x]	[ ]	Eukaryotic cell lines
[x]	[ ]	Palaeontology and archaeology
[ ]	[x]	Animals and other organisms
[x]	[ ]	Clinical data
[x]	[ ]	Dual use research of concern

Methods

n/a	Involved in the study	
[x]	[ ]	ChIP-seq
[x]	[ ]	Flow cytometry
[x]	[ ]	MRI-based neuroimaging

Antibodies

Antibodies used	secondary donkey anti-rabbit Alexa Fluor 647 (ThermoFisher, Cat#A-21449), rabbit anti-RFP (Rockland Immunochemicals,

	Cat#:600-401-379), rabbit anti-Fos (Synaptic Systems, Cat#:226 003)
Validation	Invitrogen (Thermofisher) antibodies undergo a two-part testing approach for target specificity verification and functional application validation. Rockland and Synaptic systems examines all antibodies through quantification and validation using techniques including: SDS-PAGE, Western blotting, ELISA, and Ligand binding assays. All antibodies used in this study was visualized and validated in appropriate tissue types using immunohistochemistry.

Animals and other research organisms

Policy information about studies involving animals; ARRIVE guidelines recommended for reporting animal research, and Sex and Gender in Research

Laboratory animals	Mouse :C57BL/6J, postnatal day 56-70
Wild animals	n/a
Reporting on sex	310 male mice were used for all experiments. Only male mice were utilized as the highest diagnose rates for autism is in boys.
Field-collected samples	n/a
Ethics oversight	All experimental procedures were approved by the Princeton University Institutional Animal Care and Use Committee and in accordance with animal welfare guidelines of the National Institutes of Health.

Note that full information on the approval of the study protocol must also be provided in the manuscript.
